# Multiomics signatures of type 1 diabetes with and without albuminuria

**DOI:** 10.3389/fendo.2022.1015557

**Published:** 2022-12-02

**Authors:** Marc Clos-Garcia, Tarunveer S. Ahluwalia, Signe A. Winther, Peter Henriksen, Mina Ali, Yong Fan, Evelina Stankevic, Liwei Lyu, Josef K. Vogt, Torben Hansen, Cristina Legido-Quigley, Peter Rossing, Oluf Pedersen

**Affiliations:** ^1^ Novo Nordisk Foundation Center for Basic Metabolic Research, Faculty of Health and Medical Sciences, University of Copenhagen, Copenhagen, Denmark; ^2^ LEITAT Technological Center, Terrassa, Spain; ^3^ Complications Research, Steno Diabetes Center Copenhagen, Herlev, Denmark; ^4^ The Bioinformatics Center, Department of Biology, University of Copenhagen, Copenhagen, Denmark; ^5^ Clinical Microbiomics, Copenhagen, Denmark; ^6^ Department of Clinical Medicine, University of Copenhagen, Copenhagen, Denmark; ^7^ Center for Clinical Metabolic Research, Gentofte University Hospital, Copenhagen, Denmark

**Keywords:** multiomics, type 1 diabetes, albuminuria, metabolomics, microbiome, lipidomics, phageome

## Abstract

**Aims/hypothesis:**

To identify novel pathophysiological signatures of longstanding type 1 diabetes (T1D) with and without albuminuria we investigated the gut microbiome and blood metabolome in individuals with T1D and healthy controls (HC). We also mapped the functional underpinnings of the microbiome in relation to its metabolic role.

**Methods:**

One hundred and sixty-one individuals with T1D and 50 HC were recruited at the Steno Diabetes Center Copenhagen, Denmark. T1D cases were stratified based on levels of albuminuria into normoalbuminuria, moderate and severely increased albuminuria. Shotgun sequencing of bacterial and viral microbiome in stool samples and circulating metabolites and lipids profiling using mass spectroscopy in plasma of all participants were performed. Functional mapping of microbiome into Gut Metabolic Modules (GMMs) was done using EggNog and KEGG databases. Multiomics integration was performed using MOFA tool.

**Results:**

Measures of the gut bacterial beta diversity differed significantly between T1D and HC, either with moderately or severely increased albuminuria. Taxonomic analyses of the bacterial microbiota identified 51 species that differed in absolute abundance between T1D and HC (17 higher, 34 lower). Stratified on levels of albuminuria, 10 species were differentially abundant for the moderately increased albuminuria group, 63 for the severely increased albuminuria group while 25 were common and differentially abundant both for moderately and severely increased albuminuria groups, when compared to HC. Functional characterization of the bacteriome identified 23 differentially enriched GMMs between T1D and HC, mostly involved in sugar and amino acid metabolism. No differences in relation to albuminuria stratification was observed. Twenty-five phages were differentially abundant between T1D and HC groups. Six of these varied with albuminuria status. Plasma metabolomics indicated differences in the steroidogenesis and sugar metabolism and circulating sphingolipids in T1D individuals. We identified association between sphingolipid levels and Bacteroides sp. abundances. MOFA revealed reduced interactions between gut microbiome and plasma metabolome profiles albeit polar metabolite, lipids and bacteriome compositions contributed to the variance in albuminuria levels among T1D individuals.

**Conclusions:**

Individuals with T1D and progressive kidney disease stratified on levels of albuminuria show distinct signatures in their gut microbiome and blood metabolome.

## 1 Introduction

Chronic kidney disease (CKD) is a major health burden with a prevalence of about 15% in the United States (1) with a record global rise of 41.5% mortality rates among CKD reported during the past 3 decades (2). Elevated albuminuria is strongly associated with end stage renal disease, cardiovascular disease, and death among CKD (3). Diabetes is the leading cause of end stage kidney disease, and about one third of individuals with type 1 or type 2 diabetes develop CKD, also referred to as diabetic nephropathy or diabetic kidney disease (2). Diabetic nephropathy progression in type 1 diabetes can be clinically characterized by stages of increasing albuminuria (a) moderately increased albuminuria (previously called microalbuminuria) (urinary albumin 30 to 300 mg/g creatinine), (b) severely increased albuminuria (macroalbuminuria or proteinuria) (>300 mg/g), (c) loss of renal function (glomerular filtration rate), and (d) finally need for kidney replacement therapy.

The intestinal microbiome constantly interacts with its host, constituting a dynamic balance and synergy, and thereby playing a role in maintaining and complementing metabolic and physiological functions (4). Studies in animal models of T1D support the hypothesis that an altered gut microbiome may lead to a “leaky” intestinal mucosal barrier, an imbalance in innate and adaptive immune systems and eventually triggering various chronic non-communicable diseases (5, 6). A low diversity of the gut microbiome is associated with dysmetabolism and (7) a state of dysbiosis is hypothesized to worsen the metabolic status of individuals with T1D (8, 9). Furthermore, a pathophysiological role of an imbalanced gut microbiota in diabetic nephropathy has been suggested (6).

A recent study has proposed a mechanism by which the gut microbiota impacts host’s insulin resistance and albuminuria development by upregulating G protein-coupled receptor 43 (GPR43) (10). Furthermore, the impact of the gut microbiota upon host’s metabolic status is not limited to direct interaction between organisms, but also through specific bacterial metabolites. In this context, it is of interest that a role of bacteria-derived phenyl sulfate has been reported to induce albuminuria in experimental models of diabetes (11). As for the plasma metabolome, few studies including our previous work (12) have reported associations between albuminuria and sphingomyelins, phosphatidylcholines (13) and unsaturated fatty acids and phospholipids (13–16).

In this framework, we previously identified differences in the gut bacteriome of T1D individuals stratified by albuminuria levels using a 16S rRNA gene marker approach (12). However, due to the modest taxa resolution provided by this method we failed to gain deeper insights into the bacteriome and phageome at species level and bacteriome functional potentials. The present study included the same study participants (T1D and HC) (12) but with high-resolution whole microbiome sequencing combined with untargeted plasma lipidomics and polar metabolite profiling to further investigate the single and multi-omics profiles, and their functional relationship. Thus, the objectives of the current study were: (i) to analyze both the taxonomical composition and functional potential of the metagenomic communities in T1D stratified by albuminuria levels, and in HC; (ii) to characterize the untargeted plasma metabolome of T1D, stratified by albuminuria levels; and (iii) to associate metabolome with metagenomic features, to investigate pathophysiological multiomic signatures of longstanding T1D with and without albuminuria.

## 2 Results

### 2.1 Characteristics of study participants

**Figure 1:**
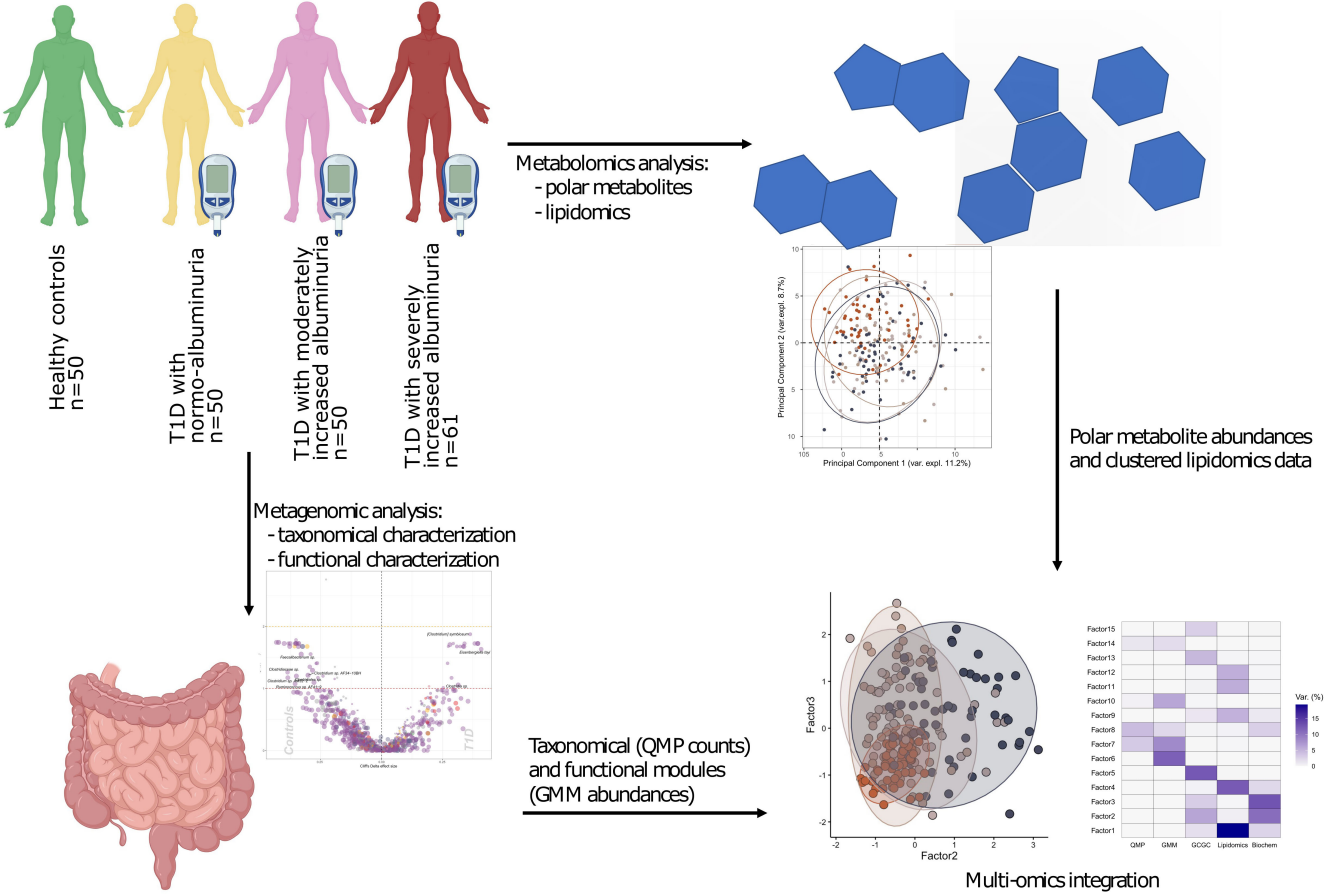
Study design and analytical overview. The study cohort comprised 50 healthy and 161 individuals with T1D, who stratified on albuminuria levels: normo-albuminuria (n=50, albuminuria levels =<3.39 mg/mmol), moderately increased albuminuria (n=50, albuminuria levels =3.39–33.79 mg/mmol) and severely increased albuminuria (n=61, albuminuria levels =≥33.90 mg/mmol). For each study participant 1) plasma samples were collected to perform non-targeted metabolomics analysis, including both polar metabolites and lipids, and 2) faecal samples collected for metagenomics analysis. For the metagenomic samples, differences between the clinical groups at both taxonomical and functional level were assessed. Lipidomics data was clustered into highly correlated lipid clusters in order to reduce dimensionality. Finally, different omics data types were integrated with the Multi Omics Factor Analysis (MOFA+) tool and metabolite origin screened to identify bacterial- and host-related metabolites through Least Shrinkage Selector Operator (LASSO) regression models. QMP, Quantitative Microbial Profiles; GMM, Gut Metabolic Modules.

The study comprised 161 T1D individuals (50 with normoalbuminuria, 50 with moderately increased albuminuria and 61 with severely increased albuminuria and 50 healthy controls (HC). A study overview is given in **Figure 1**.

The study participants were aged 60 ± 11 years (mean ± SD), 42% being women. The mean diabetes duration was 42 ± 15 years with an eGFR of 75 ± 25 ml min^−1^ (1.73 m)^−2^ among individuals with type 1 diabetes (T1D). Detailed clinical characteristics of the study groups have been reported (12) and are again presented in **Supplementary Table 1**.

T1D individuals with elevated albuminuria were treated with anti-hypertensive and proton pump inhibitor drugs more frequently than others (**Supplementary Table 1**). HbA1c, and fasting plasma hs-CRP levels were expectedly higher and hemoglobin, and fasting plasma concentrations of total cholesterol and LDL cholesterol were lower in the T1D individuals upon stratification for increasing albuminuria and when compared to HC (**Supplementary Table 1**).

Serum creatinine was higher and corresponding eGFR levels lower in moderate and severely increased albuminuria groups compared to T1D with normoalbuminuria. The Bristol stool scale score and estimated bowel movement frequency were comparable in each T1D albuminuria group. The daily dietary macronutrient intake differed significantly between the albuminuria groups as described previously (12).

### 2.2 Community, taxa, and functional modules of the gut bacterial microbiota

In total 9,229 genes were mapped for the gut bacteriome using KEGG Orthology (KO) annotation. The gene richness distribution (**Supplementary Figure 1**) and the alpha (or intragroup) diversity (**Supplementary Figure 2**) were not distinctive of the four study groups when using the rarefied data (QMP count; **Supplementary Figure 3**; **Supplementary Table 2**), except when using Shannon index (p = 0.02). However, pairwise comparison showed lower diversity in moderately and severely increased albuminuria groups compared to controls when using Shannon (p_micro_= 0.015 and p_macro_ = 0.004), or Simpson (p_micro_= 0.08 and p_macro_= 0.02) indices (**Supplementary Figure 2**).

The community bacteriome dispersion varied between T1D and HC (p_adonis_=0.001). These differences were mainly driven by T1D individuals with moderately and severely increased albuminuria groups (p_permanova_ = 0.006), respectively. Furthermore, the T1D individuals were heterogeneously dispersed on the PCoA plot (Jensen-Shannon divergence index) compared to controls (**Supplementary Figure 4**). These observations were consistent with the 16s rRNA gene marker results previously reported by Winther et al. (12).

Shotgun sequencing allowed us to annotate the bacterial taxonomy to species level (Metagenomic species or MGS). Taxonomical annotations are provided in **Supplementary Table 3**.

**Figure 2:**
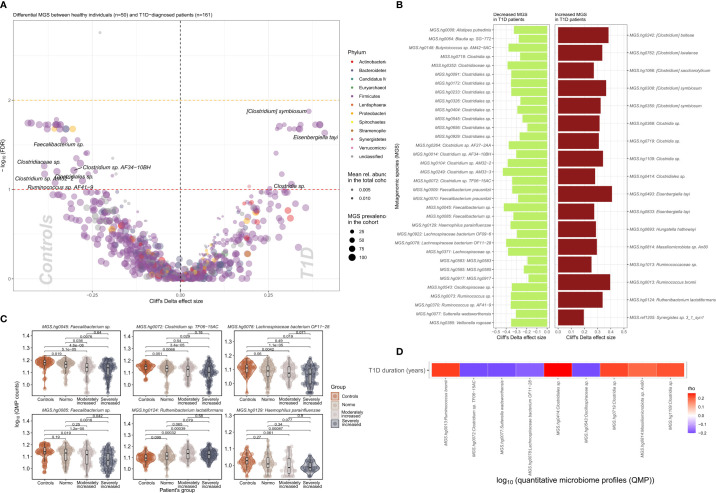
Differential bacterial abundance at metagenomic species (MGS) level, between T1D and healthy individuals. **(A)** Volcano plot showing difference in absolute abundance of metagenomic species (MGS) between T1D individuals (n=161) and the healthy controls (n=50). X-axis indicates Cliff’s Delta effect size; Y-axis represents FDR-corrected (negative log) p–values. MGS that associated positively with T1D have been depicted towards increasing direction of effects (right). MGS circle color depicts the corresponding annotated phylum. Circle size corresponds to the number of individuals in the cohort where the specific MGS was found. Transparency of the circle corresponds to the average relative abundance in which each MGS is found within participants. **(B)** Significant contrasts in MGS abundance between T1D and healthy controls depicted with the bar length corresponding to Cliff’s Delta effect size: green for higher and red for lower MGS abundances within T1D individuals. **(C)** Differential absolute MGS abundance between T1D subgroups stratified on levels of albuminuria. Individual distribution of the log10 transformed QMP counts (absolute abundance) is depicted for each MGS in violin and dot plots. Global distribution of the MGS counts for each T1D subgroup is depicted with a boxplot, indicating median value of the distribution with a horizontal line, first and third quartile with the limits of the white rectangle and the upper and lower limits of the distribution with vertical bars. Significance for pairwise comparison between different study groups is indicated with p-value. **(D)** Significant correlations between T1D duration in years and absolute abundance of MGS (QMP counts) are depicted as a heat map. Positive correlations are shown in red and inverse correlations in blue.

We identified 51 bacterial metagenomic species (MGS, hereafter called species) that were differentially abundant between T1D and HC (**Figures 2A, B**; **Supplementary Table 4**) where the absolute abundance of 17 species were higher while 34 were lower in T1D individuals. In general lower absolute abundance of Short-Chain Fatty Acids (SCFA) producers such as *Veilonella rogosae* (17)*, Faecalibacterium* sp.*, Butyricicoccus* spp. *Clostridiales* sp. and *Lachnospiraceae bacterium* was observed in T1D compared to HC. *Clostridium* spp., including *C. saccharolyticum*, known for its saccharolytic activity in addition to *Eisenbergiella tayi, Hungatella hathewayi* and *Ruthenibacterium lactatiformans* were more abundant in T1D (**Figures 2C, D**). Some of the bacterial taxa (MGS) differences observed between overall T1D and HC individuals were also observed between T1D individuals with moderate or severe albuminuria and HC individuals (**Figure 2C**; **Supplementary Figures 5, 6**). **Supplementary Figure 7** provides an overview of MGS absolute abundance within the T1D albuminuria subgroups and MGS specific and common to moderate and severely increased albuminuria groups, compared to HC.

Further shotgun sequencing of the metagenome facilitated mapping of functional metabolic potential and anaerobic fermentation capacity of the metagenome in form of Gut Metabolic Modules (GMMs) curation that represent a cellular enzymatic process defined by input and output metabolites. The GMMs computed using the Omixer Reference Pathway Mapper and KEGG Orthology (RPM) (18) differed in abundance (**Supplementary Table 5**) in T1D group compared to HC using univariate analyses (**Supplementary Table 6**; **Supplementary Figure 8**). The T1D bacteriome was enriched for modules of sugar degradation (most abundantly for ribose, fucose and trehalose) and for modules of amino acid metabolism, particularly for non-polar amino acids (alanine, glycine, isoleucine, methionine, and tryptophan), followed by acidic (lysine, cysteine, and histidine) and polar (threonine and glutamine) amino acids (P_FDR_<0.10; **Supplementary Figure 8**; **Supplementary Table 6**).

### 2.3 Gut phageome and type 1 diabetes

**Figure 3:**
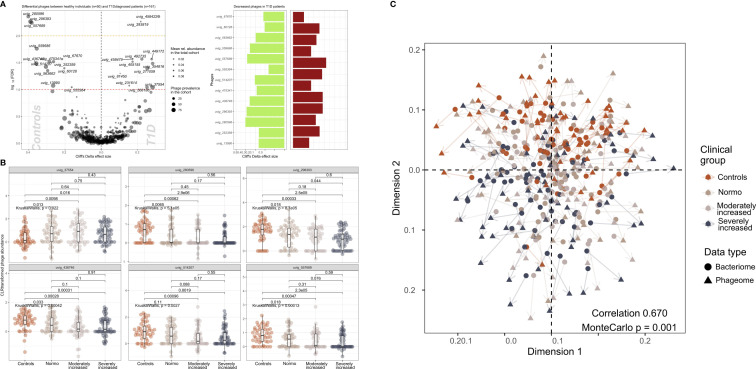
Contrasted relative abundance of bacteriophages in T1D and healthy control individuals. **(A)** In the left panel, a volcano plot compares the relative phage abundances between T1D and healthy individuals. The X-axis represents Cliff’s Delta effect size while the Y-axis represents the association threshold for the comparison, (negative log) FDR-adjusted p-value. Significantly contrasted phages (above the red dotted line) are annotated. Dot size is relative to the global prevalence of phage in the present study sample, while their transparency is relative to their mean abundance in the cohort. In the right panel, significantly contrasted phages are shown as bar plots corresponding to Cliff’s Delta effect sizes. Phages with significantly lower abundance in T1D are indicated as green bars while red bars represent phages with significantly higher abundance in T1D. **(B)** Distribution of abundance of selected phages when comparing healthy controls with the three groups of T1D stratified by albuminuria. For each phage, the CLR-transformed abundance distribution is represented in differently colored dots and boxplots showing the global group distribution. Multi-group comparison computed with Kruskal-Wallis test is included in each plot, as well as the pairwise comparisons between all study groups, performed with Wilcoxon test. **(C)** Comparison between the global composition of the phageome and the bacteriome, performed with Procrustes test. Principle coordinates analysis (PCoA) shows the disposition of individuals in the corresponding scores plot, for both bacteriome (circles) and phageome (triangles) based distance matrix. An arrow has been drawn connecting the same individuals. Individuals have been colored depending on their clinical group. Correlation and significance are indicated in the bottom right corner of the plot.

We identified 502 highly abundant phages in our study participants of which 25 were differentially enriched between T1D and HC (**Figure 3A**). Interestingly, the relative abundance of six of these differentially enriched phages (uvig_37554, uvig_280596, uvig_296393, uvig_436746, uvig_514207, uvig_557689) changed with increasing level of albuminuria (**Figure 3B**).

Furthermore, CLR-normalized phage abundances were associated inversely with multiple clinical factors (adjusted for age, sex, and diet) including T1D status (25 phages), diabetes duration (20 phages), eGFR (21 phages) and plasma creatinine (7 phages) levels (**Supplementary Figure 9**). Hematocrit was found to be related to 17 phages overlapping with hemoglobin and partly glycosylated hemoglobin. All these associations were partly overlapping with T1D-associated phages (**Supplementary Figure 9**). The distribution of the samples derived from the bacteriome and the phageome analyses were similar, as assessed by Procrustes analysis (correlation = 0.67; **Figure 3C**).

### 2.4 Plasma metabolome and lipidome

#### 2.4.1 Plasma polar metabolites

We examined the differential abundance of 398 (143 known and 255 unannotated) plasma polar metabolites (**Supplementary Table 7**) between i) T1D versus HC and, ii) albuminuria subgroups within T1D, using univariate and multivariate approaches.

**Figure 4:**
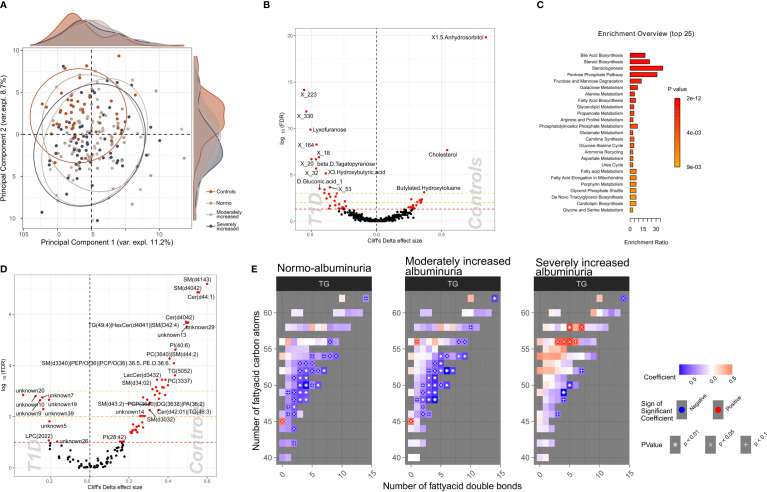
Profiles of plasma polar metabolites and lipids in T1D and healthy control individuals. **(A)** Scores plot resulting from the partial least square discriminant analysis (PLS-DA) demonstrating polar metabolites that contribute highly (Variable influence on project (VIP) > 1) to a T1D signature. Colors depict clinical study groups and ellipses demarcate the spread of individuals within each group. Explained variance for first two principal components (PC1 and PC2) is indicated on the respective X- and Y- axis. Global distribution of the participants from each clinical group is shown as a density plot in the secondary Y-axis. **(B)** Univariate analysis (volcano plot) depicting differential circulating polar metabolite abundances between healthy and T1D individuals. Significantly contrasted (FDR < 10%) polar metabolites are depicted in red, with increasing effects in direction of controls (right). **(C)** Functional metabolic pathway identification comparing T1D and healthy controls and depicting enriched metabolic pathways: annotations of top polar metabolites using the human metabolome database (HMDB). Bars have been ordered and colored by the enrichment p-value score. **(D)** Univariate analysis (volcano plot) depicting differential abundances of serum lipid cluster between healthy and T1D individuals. Significantly contrasted (FDR < 10%) lipid clusters are colored in red with increasing effects in direction of controls (right). **(E)** Triglyceride (TG) species associated with different T1D albuminuria groupings (compared to healthy controls) based on the number of carbon atoms (Y-axis) and the number of double bonds (X.axis) present in the chemical structure. Blue-to-red gradient heatmap suggests directionality of the association (negative to positive). Significant associations are indicated in each cell with the following symbols: + p<0.1, X p<0.05, *p<0.01.

To identify a subset of T1D - linked metabolites, we used the partial least squares-discriminant analysis (PLS-DA) approach (multivariate) splitting the dataset into 70% training and 30% validation sample subsets. Albeit we achieved a good separation (R^2^: 91.8%) using five components, the reproducibility of the model was limited (Q^2^: 25%; **Supplementary Figure 10**). Next, we selected polar metabolites (n=132) with a VIP (Variable Importance in Projection score) ≥ 1 in the PLS-DA analysis and we generated a PCA plot that effectively differentiated between T1D and HC groups, whereas the score did not provide any differentiation between albuminuria groups. (**Figure 4A**).

We identified 58 polar metabolites that were differentially abundant between T1D and HC (P_FDR_<10%; **Figures 4B, C**; **Supplementary Table 8**) using the univariate approach. In T1D individuals, the plasma concentration of 1,5-anhydrosorbitol was significantly lower followed by cholesterol and butylated-hydroxytoluene while several sugar derived metabolites like lyxofuranose and beta-D-tagatopyranose were higher (**Figure 4B**; **Supplementary Table 8**). Within the T1D group a univariate comparison between moderately and severely increased albuminuria groups revealed significantly (P_FDR_<10%) lower levels of ribitol, benzeneacetic acid, decanoic acid and 3-phenylpropanoic acid while higher levels of 2,3-dihydroxybutanoic acid in the severely increased albuminuria group (**Supplementary Figure 11**).

#### 2.4.2 Functional enrichment of plasma polar metabolites and metabolic pathways

The functional enrichment analyses of plasma polar metabolites (T1D vs. HC) led to identification of several enriched human metabolic pathways. The differentially enriched pathways mainly comprised of steroidogenesis and steroid biosynthesis, bile acid biosynthesis, pentose phosphate pathway and several sugar metabolism pathways, such as fructose and mannose degradation and galactose metabolism (**Figure 4C**; **Supplementary Table 9).**

#### 2.4.3 Plasma lipidomics

7,470 plasma lipids (476 known and 6,994 unannotated, **Supplementary Table 10**) were clustered into 122 strongly correlated lipid clusters ranging between 3 to 1,054 lipids per cluster (**Supplementary Tables 11, 12**).

We identified 60 lipid clusters (P_FDR_<10%) differentially abundant between T1D and HC (**Supplementary Table 13**). The T1D lipidome was enriched in a set of lysophosphocholines (LPCs) and unknown lipids containing 20 to 22 carbon atoms (**Figure 4D**). Inversely, lipid clusters containing long chain ceramides (40-44 carbon atoms) and sphingomyelins (30-41 carbon atoms) were highly abundant in HC compared to T1D individuals.

Further, we analyzed the distribution of the annotated lipidome stratified by albuminuria status within the T1D and compared these to HC. Triglyceride (TG) lipid species with large number of carbon atoms (>55 carbon atoms) were positively associated with severely increased albuminuria, while comparatively shorter TGs (40-55 carbon atoms) were inversely associated to both normo-albuminuria and moderately increased albuminuria groups (**Figure 4E**).

### 2.5 Multi-omics factor analysis based on albuminuria levels

Multi-Omics Factor Analysis 2 (MOFA2) (19) tool allowed us to integrate all data on gut microbiome, plasma metabolites (metabolomics and lipidomics), and clinical biochemistry and, by a process of factorization, identify which data type was the most contributing to the individuals’ T1D phenotype (stratified on albuminuria status).

**Figure 5:**
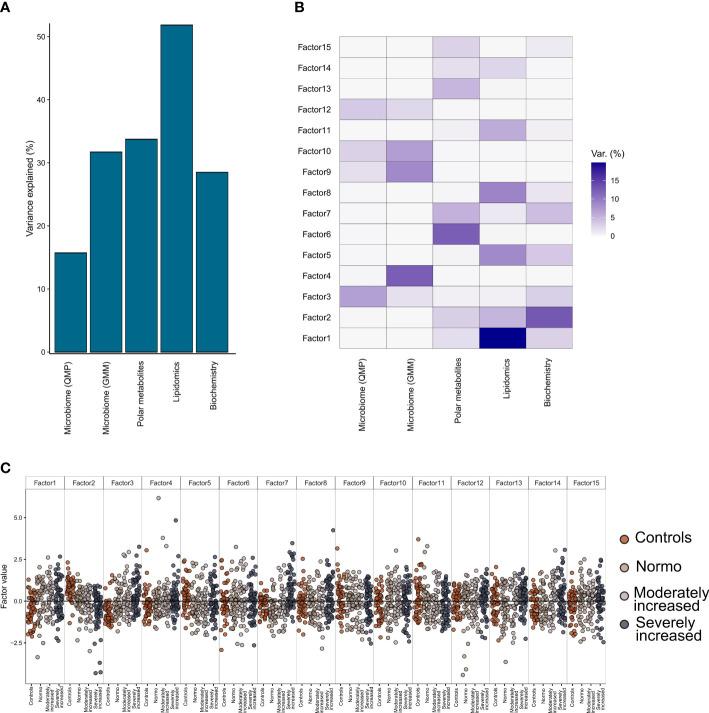
Multi-Omics Factor Analysis (MOFA). Results from multi-omics data integration after combining multiple data types: metagenomics data (taxonomical and functional), plasma metabolomics data (polar metabolites and clustered lipids) and biochemistry data using the MOFA+ tool. **(A)** Global explained variance for each of the data types (included in the integration step) has been represented as a bar chart. **(B)** Composition of 15 individual factors generated with the proportion of explained variance by each of the data types, displayed as a white-to-blue gradient in increasing order. **(C)** Distribution of the eigenvalues obtained for each of the factors for the combined dataset. Within each of those factors the individual, eigenvalues are represented as dot plots. Different colors in the dot plot depict different clinical study groups: orange for healthy controls, dark gray for T1D with normo-albuminuria, light gray for T1D with moderately increased albuminuria and black for T1D with severely increased albuminuria. Each of the study groups are also labelled in the bottom horizontal axis.

From the factorization results, we observed that the lipidomics dataset explained a major part of the factors composition (~50% variance), followed by the polar metabolites, bioclinical variables, and the functional bacterial profiling (GMM) of the gut microbiome (~30% of factor’s variance) (**Figure 5A**). Finally, analysis of the taxonomical composition of the bacteriome (QMP) explained about 15% of the factors’ composition. Furthermore, we observed a rather limited interaction between the plasma metabolome and the gut microbiome data, as no factors were found that combined effectively these two data types (**Figure 5B**). Instead, the combination of biochemistry analysis and polar metabolites (factors 2 and 3) differentiated well between T1D and HC individuals. Thus, by splitting all the data combined into 15 factors we observed a trend by which T1D status (and partly albuminuria levels) influenced the position of the individuals along the generated principal components (**Figure 5C**).

Details on clinical and metabolomic components resulting in Factors 2 and 3 have been presented in (**Supplementary Figure 12**).

#### 2.5.1 Relationships between the gut bacteriome and differential blood metabolome

**Figure 6:**
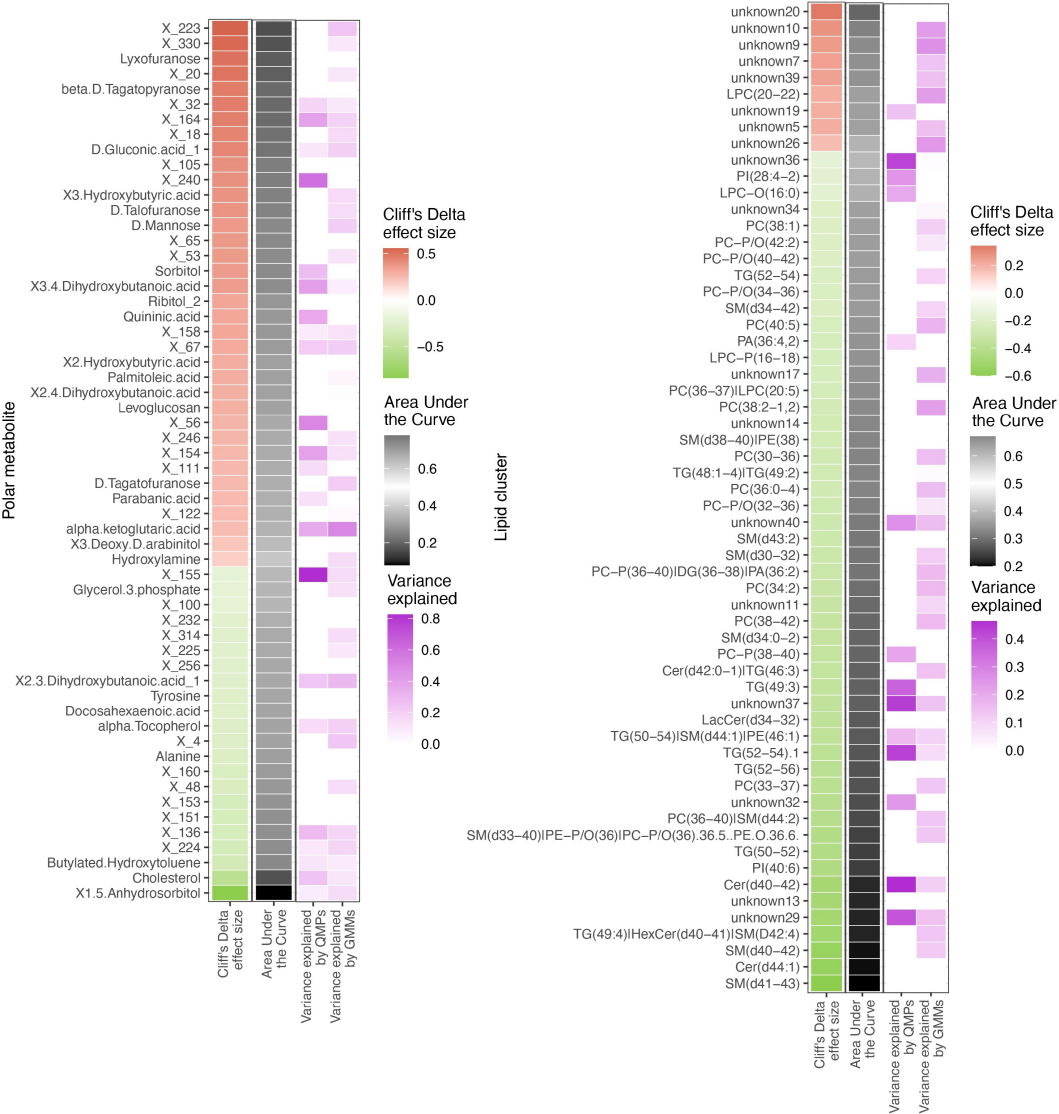
Differentially abundant circulating metabolites and microbiome origin and functionality assessment. Summary of the circulating metabolites and lipids data integrated with gut microbiome origin and functionality in the T1D vs healthy controls comparison. The two heatmaps display significantly contrasted (FDR < 10%) polar metabolites (left) and lipid clusters (right) between T1D and healthy individuals. Column 1 (red green of each of the two heatmaps shows the Cliff’s Delta effect size for the clinical group comparison, ordered from higher to lower abundance in T1D individuals and colored from red (higher) to green (lower) gradient depending on its relative abundance in T1D individuals. Column 2 (black-white) shows the usefulness of the metabolite for discrimination between T1D and healthy individuals based on Area Under the Curve (AUC) analyses. The black-to-white-to-black gradient for AUC depicts assessment ability with 50% being uninformative AUC, 0% being the limit for the identification of healthy individuals and 100% being the limit for identification of T1D individuals. Column 3 (purple white) depicts relationship between the metabolites and bacterial QMP counts (absolute MGS abundance). Cells are colored if there is any significant association with metabolite levels (based on LASSO modelling) and the white-to-purple color gradient depicts the explained variance by the microbiome. Column 4 (purple white) depicts the relationship between the circulating metabolites and functional abundance of GMMs. Cells are colored if there is any significant association with metabolite levels (based on LASSO modelling) and the white-to-purple color gradient depicts the explained variance by the microbiome metabolic potential (or functionality).

Since metabolome composition explained the factors composition better than the other data types, we assessed the relationships of the gut bacteriome and the blood metabolome (**Figure 6**). Overall, 30% of the metabolites were associated with the bacteriome taxonomical profiling, while 40% to 50% of it was related to the bacteriome *via* functional profiling (**Supplementary Figure 13**)

All the differentially abundant polar metabolites in the T1D individuals were associated to the bacterial abundances of *Faecalibacterium prausnitzii, Clostridium spps.*, Lachnospiraceae spp. and *Eisenbergiella tayi*.

The lipidome composition was mostly associated with the bacteriome functional profiling (GMMs). In addition, the lipidome composition was associated to only a small subset of specific bacterial and/or archaeal species, such as *Akkermansia muciniphila* and *Methanobrevibacter smithii.*

## 3 Discussion

In the present study, applying deep metagenomic sequencing-based functional annotation and multi-omics factorization we identified multiple additional molecular signatures for T1D in the gut microbiome and plasma metabolome and lipidome both individually and when combined, compared to our previous findings (12). Moreover, we provide a gut phageome profile for T1D. While most significant differences in the gut microbial abundance and circulating metabolites and lipids were observed between T1D and HC, both moderately and severely increased albuminuria groups also evidenced significantly enriched bacteriome and plasma metabolite levels when compared to HC. In functional bacteriome analyses, we identified sugar, amino acid, and short chain fatty acid (SCFA) metabolizing species differentially enriched in T1D compared to HC, while no significant differences in the functional nature of the bacteriome was observed upon albuminuria stratification. The latter may suggest that nephropathy development is a continuous process, modifying both the microbiome and the metabolome in a constant manner. Through multi-omics we report that circulating lipids explained most of the phenotypic variance for T1D (stratified for albuminuria) followed by polar metabolites, clinical factors, and functional gut bacterial profiling (GMM). Albeit a limited interaction between circulating metabolome and gut microbiome was observed, a combination of circulating polar metabolites and clinical risk factors could best differentiate between T1D and HC individuals.

Deep shotgun sequencing of the microbiome allowed us to annotate the bacterial taxonomy to species or strain level (Metagenomic species or MGS) facilitating mapping of functional metabolic potential and anaerobic fermentation capacity of the metagenome in form of specie-function relationship or Gut Metabolic Modules (GMMs) (18). The absolute number of differentially abundant MGS were relatively higher in the group of severely increased albuminuria, followed by moderate and reduced in normo-albuminuria groups within the T1D cases (**Supplementary Figure 7**). Overall, a higher number of MGS seemed to occur with a lower absolute abundance in the T1D group compared to HC (**Figure 2B**). This is not surprising as most immune disorders have been generally associated with a loss of gut microbial diversity, specifically *Akkermansia* and *Faecalibacterium*, both potentially contributing to host immune tolerance (20). We also report significantly lower absolute abundance of *Faecalibacterium* in the T1D group compared to HC.

Alteration of the gut microbiota in T1D resulted in lower SCFA production capabilities and increased saccharolytic activity. SCFAs are carboxylic acids with aliphatic tails of 1-6 Carbon atoms, acetate (C2), propionate (C3) and butyrate (C4), being most abundant produced by anerobic fermentation of polysaccharides or dietary fibers. SCFAs are mainly produced by *Bacteroidetes* (C2 and C3) and *Firmicutes* sp. (C4) further promoting beneficial bacteria survival (21, 22). Dietary fiber can upregulate carbohydrate metabolism enzymes, increasing SCFAs that enhance intestinal epithelial barrier function (particularly C4) reducing metabolic toxins and expression of inflammatory molecules (23). The C4 SCFA is also deemed important for hypoxia inducible factor 1 (HIF1) stability in maintaining epithelial barrier through C4-based oxygen balance and maintaining low oxygen concentrations in gut (21). A recent study also reported that lower SCFAs were associated with metabolic syndrome (22) and autoimmune disorders like inflammatory bowel disease (21). The absolute abundance of the majority of *Clostridiales* sp. belonging to phylum Firmicutes was significantly lower in the T1D in the current study. A polysaccharide treatment induced increase in abundance of SCFA producing *Clostridiales* sp. has been reported to lower blood glucose levels, improve glucose tolerance and restore lipid balance in a rat model of type 2 diabetes (24) further supporting our findings and the relationship between lower bacterial abundance, correspondingly lower bacteriome function and T1D. The absolute abundance of *R. lactatiformans*, a lactate-producing bacterium (25), was also higher in T1D partially aligning with the elevated blood lactate levels recently reported in T1D (26). In addition, the absolute abundance of *Clostridium Spp.*, which is known for its sugar degradation capabilities was higher in the T1D group. Interestingly, abundance of *H. hathewayi*, which is reported to be positively associated with circulating taurine levels (27) was enriched in T1D. Given the fact that taurine levels reduce hyperglycemia (28, 29), abundance of *H. hathewayi* among T1D may explain the body’s compensatory mechanism to counter hyperglycemia.

Functional classification of the T1D associating plasma metabolites suggested pathways enriched for steroidogenesis, bile acid biosynthesis and sugar metabolism. Additionally, the T1D plasma lipidome profile associated with alterations in the circulating sphingolipid (SL) levels, especially ceramides, which were partially produced by the gut microbiota, particularly Bacteroides sp. (**Supplementary Table S14**). Sphingolipids are known for their bioactive role as secondary messengers especially in metabolic disorders. Recently, it has been demonstrated that gut bacterial sphingolipids may pass the intestinal epithelium barrier, modifying the host’s sphingolipid metabolism (30). Particularly, bacterial sphingolipids inhibit the processing of the host’s own sphingolipids, including ceramides, the lipid type most altered in T1D. In the current study, lipids, especially long chain sphingolipids (ceramides and sphingomyelins) were multifold lower in T1D compared to HC, irrespective of the albuminuria stratifications. Recent studies supporting our findings observed associations between host circulating long chain ceramides and reduced kidney function (13) and diabetic kidney disease (14, 15) in T1D. We recently reported long chain sphingomyelins, to be inversely associated with albuminuria (especially severely increased albuminuria vs. normoalbuminuria) in 669 individuals with T1D (13) which were also observed in the DCCT/EDIC trial (15) but remained inconclusive in the current study potentially due to limited sample size. Animal studies have confirmed the role of sphingolipids-derived ceramides in insulin resistance of liver (31) while their pharmacological inhibition improved glucose homeostasis (32). However, the mechanism by which sphingolipids may influence albuminuria development is unclear. Bacteria-derived sphingolipids have been reported to act as ligands for G protein-coupled receptors (33), including those found in the intestinal epithelium (34, 35). These results are compatible with the albuminuria-inducing gut microbiota action hypothesis mediated through GPR43 upregulation (10).

The observed metabolomics profile was in accordance with our previous study looking into the pathophysiology of diabetic kidney disease (DKD) (36). In the present study we identified several associations between plasma metabolites and albuminuria in T1D. The main association was with polyols where we showed that plasma concentrations of sorbitol and ribitol both were higher in direct proportion to higher levels of albuminuria. The same metabolites, pointing to a validation in an independent cohort, applied to hydroxy butyrate 3,4-dihydroxybutanoic acids which were associated with moderate and severely increased albuminuria and have now replicated in the present study. Most interestingly sorbitol 3,4-dihydroxybutanoic and quininic acid were among the three identified metabolites showing strong associations to the abundance of bacterial species (**Figure 2**). However, the outcome of the functional enrichment results of circulating metabolites in T1D group can mainly be characterized as alterations related to two metabolic functions: cholesterol biosynthesis, and glucose metabolism.

The altered cholesterol levels resulted in a specific enrichment of steroid hormones biosynthesis and related metabolic pathways. Alterations of sexual hormone levels, with link to reduced fertility and increased risk for cardiovascular disease have been previously reported in T1D (20), which might be related to our results and the specific steroid metabolic alterations. For the glucose metabolism, the pentose phosphate metabolic pathway was highly enriched in our dataset. Interestingly, a protective role against chronic diabetes complications, including diabetic nephropathy, has been previously reported for this metabolic pathway (21, 22, 37).

Further factorization of multi-omics data in the current study demonstrated that the circulating lipidome could explain most of the (50%) T1D phenotypic variance followed by polar circulating metabolites, functional bacterial profiles (GMM) and finally 15% by taxonomical bacterial composition (QMP) (**Figure 5**). However, no distinction based on level of albuminuria could be made. We identified two distinct interactions that differentiated the T1D and HC groups best. These factors (factor 2 and 3, **Figure 5**) comprised a set of polar metabolites and bio-clinical markers. Interestingly the most significant drivers within these clinical and metabolite features were diabetes duration and a combination of sugar derivatives (**Supplementary Figure 12**). However, only a limited interaction between plasma metabolome and gut microbiome data was evidenced. While investigating metabolome origins we found polar metabolites that were associated with bacterial abundance reflected in relevant GMMs enriched for amino acids metabolism. Similarly, the lipidome was associated with bacterial function through relationships between the lipidome composition and abundance of GMMs involved in sugar degradation.

On this note, we need to consider the role of medication in both the gut microbiome (38, 39) and the blood metabolome (40), especially considering the amount of time T1D individuals have received medication (30 to 45 years since diabetes diagnosis). We observed a strong influence of statins on abundance of *Clostridia* and *Ruminococcaceae spps.*, while the abundance of remaining gut bacteria seemed less influenced by the prescribed drug regimes.

Our study doesn’t come without limitations, the most obvoius fact being this is a cross-sectional study, which limits the evaluation of the gut microbiome's contribution to albuminuria development. The findings reported here are based on bioinformatics analysis of combined omics technologies, which is a recent research field lacking standardized protocols, hampering reproducibility. The same holds for the metabolomics data analysis, which could only annotate a modest fraction of the identified metabolites. Moreover, identification of phages in the current study was limited to annotations available in the Gut phage database (19). Finally, the findings reported here are based on one specific cohort. Considering that the microbiome and the metabolome are also affected by a combination of environmental and biological factors, a validation on a different cohort with different environmental conditions would provide with more robust results.

Gut microbiome composition is known to be modifiable by several environmental factors, such as diet (41) or exercise (42) and/or through fecal microbiota transplant (FMT). Since this study demonstrates a relationship between the dysbiotic gut microbiota and an altered plasma metabolome composition in T1D cases with albuminuria, and if our findings are replicated in independent studies, it might serve as a basis for future microbiota-based interventions in T1D with albuminuria. Such interventions might include diet modifications or prescription of second-generation probiotics.

## 4 Materials and methods

### 4.1 Study design

A cross-sectional study conducted during 2016-2017 recruited 161 type 1 diabetes (T1D) individuals followed at the Steno Diabetes Center Copenhagen (SDCC) outpatient clinic and 50 non-diabetic age and gender matched healthy control individuals (12). All participants were >18 years of age and type 1 diabetes was diagnosed according to the WHO-criteria. Exclusion in the current study participation involved presence of at least one of the following conditions, (a) non-diabetic kidney disease; (b) renal failure (estimated glomerular filtration rate or eGFR <15 ml min^−1^[1.73 m]^−2^), dialysis or kidney transplantation; (c) change in renin–angiotensin–aldosterone system (RAAS)-blocking treatment during the month prior to study inclusion; (d) treatment with systemic antibiotics in the 3 months prior to recruitment; and (e) treatment with systemic immunosuppressive agents. Individuals with T1D were stratified into three different albuminuria groups based on the highest urine albumin/creatinine ratio (UACR) level measured on the study visit or documented previously in two out of three consecutive urine samples within past 1 year (as 24 h urine albumin content in samples [UAER] or UACR). Basis albuminuria groupings, 50 individuals had normoalbuminuria (<3.39 mg/mmol corresponding to <30 mg/24 h or mg/g), 50 had moderately increased albuminuria (3.39–33.79 mg/mmol corresponding to 30–299 mg/24 h or mg/g) and 60 had severely increased albuminuria (≥33.90 mg/mmol corresponding to ≥300 mg/24 h or mg/g). There was no recorded history elevated albuminuria for participants classified as having normoalbuminuria. For the severely increased albuminuria group, at least 30 individuals were selected based on concurrent eGFR <60 ml min^−1^[1.73 m]^−2^. The study design has been described in **Supplementary Section** (**Figure 1**, Supp Text). The study was conducted in accordance with the Declaration of Helsinki and approved by the Ethics Committee of the Danish Capital Region (protocol H-15018107). All participants gave written informed consent and provided with self-collected fecal sample for posterior metagenomics analysis (**Figure 1**).

### 4.2 Metagenomics

#### 4.2.1 Sequencing

Sequencing and metagenomic species (MGS) generation was performed as previously described (43). Quality control of raw FASTQ files was performed using KneadData (v. 0.6.1) to remove low-quality bases and reads derived from the host genome as follows: Using Trimmomatic (v. 0.36), the reads were quality trimmed by removing Nextera adapters, leading and trailing bases with a Phred score below 20, and trailing bases in which the Phred score over a window of size 4 drops below 20. Trimmed reads shorter than 100 bases were discarded. Reads that mapped to the human reference genome GRCh38 (with Bowtie2 v. 0.2.3.2 using default settings) were discarded. Read pairs in which both reads passed filtering were retained; these were classified as high-quality non-host (HQNH) reads.

#### 4.2.2 Metagenomic species generation

As reference gene catalogue, we used the Clinical Microbiomics Human Gut 22M gene catalogue (22 459 186 genes), which was created from >5000 deep-sequenced human gut specimens. For MGS abundance profiling, we used the Clinical Microbiomics HGMGS v.2.3 set of 1273 MGS, which has highly coherent abundance and base composition in a set of 1776 reference human gut samples (44).

HQNH reads were mapped to the gene catalogue using Burrows-Wheeler Alignment (BWA) men (v. 0.7.16a) with options to increase accuracy (-r 1 -D 0.3). PCR/optical duplicates were removed using samtools (v. 1.6). Each individual read was considered mapped if the following criteria were met: an alignment of ≥ 100 bases, ≥ 95% identity in this alignment, and a mapping quality (MAPQ) ≥ 20. However, if a read failed to align to the gene sequence with > 10 bases at either end, it was considered unmapped. Reads meeting the alignment length and identity criteria but not the MAPQ threshold were considered multimapped. Reads failing the alignment length or identity criteria were considered unmapped. Read pairs were classified into one of three possible categories as follows:

1) Read pairs in which both individual reads were considered unmapped.
2) Read pairs in which both individual reads were multimapped, or were mapped to genes in different MGSs, or one was multimapped and the other was unmapped, were considered multimapped.
3) Read pairs in which both individual reads mapped to the same gene; or in which one read mapped to a gene and the other was unmapped, multimapped, or mapped to another gene in the same MGS (see below); were considered mapped. A gene counts table was created with the number of mapped read pairs (for each gene), unmapped read pairs, and multimapped read pairs.

For each MGS, the “core” genes were defined as the 100 genes specific for the MGS and with the highest correlation to the mean and lowest absolute deviation from the mean. A MGS counts table was created based on the total gene counts for the 100 core genes of each MGS. However, MGS was considered detected only if read pairs were mapped to at least three of its 100 core genes; counts for MGSs that did not satisfy this criterion were set to zero. The MGS counts table was normalized according to effective gene length (accounting for read length) and then normalized to sum to 100%, resulting in relative abundance estimates of each MGS. Down-sampled (rarefied) MGS abundance profiles were calculated by random sampling, without replacement, from each sample in the MGS counts table. Values with fewer than three counts after down-sampling were set to zero, and the counts table was normalized according to effective gene length and then normalized to sum 100%.

#### 4.2.3 Computation of quantitative microbial profiles

Faecal samples were subjected to bacterial cell counting with flow cytometry. Aliquots of 0.08-0.15 g defrosted faeces were diluted 2,118 times in staining buffer (1 mM EDTA (*Sigma-Aldrich*), 0.01% Tween20 (*Sigma-Aldrich*), pH 7.2 DPBS (*Lonza BioWhittaker*), 1% BSA (*Sigma-Aldrich*)). In order to remove debris from the faecal solutions, samples were filtered using a sterile syringe filter (pore size 5 μm (*pluriSelect*)). Next, 170 μL of the bacterial cell suspension was stained with 20 μL DAPI (1mM in H_2_O, *Sigma-Aldrich*). The flow cytometry analysis of the bacterial cells present in the suspension was performed using a BD Fortessa LSRII flow cytometer (*BD Biosciences*). Measurements were performed at a pre-set flow rate of 0.5 μL/sec. Fluorescence events were monitored using the 440/40 nm, 575/26 nm, and 695/40 nm optical detectors, respectively. Forward and sideways-scattered light was also collected. The BD FACSDiva™ Software was used to gate and separate the bacterial fluorescence events from the faecal sample background. A threshold value of 900 was applied on the area of forward scattered channel (FSC) and a threshold value of 200 was applied on the area of sideways scattered (SSC) channel. Other flow settings are listed in **Supplementary Table 16**.

Density plots of blue fluorescence (440/40 nm) versus FSC allowed for distinction between the stained microbial cells and instrument noise or sample background. Density plots of red fluorescence (695/40 nm) versus FSC allowed for distinction between the counting beads and other particles in the testing solution, including bacteria, instrument noise or sample background. The exact same gates and gating strategy were applied for all samples in the form of a fixed template to allow direct comparison between measured samples.

Bacterial cell counts were later used for quantitative microbial profiling (QMP), as described elsewhere (45). Briefly, data was rarefied to equal sampling depth and cell counts used to compute the total abundance of each MGS.

#### 4.2.4 Computation of gut metabolic modules

Emapper software (v. 1.0.3, HMM mode) was used to compare each gene in the gene catalogue to the EggNOG (v. 4.5) orthologous groups database (http://eggnogdb.embl.de/), resulting in annotations for 65% of genes. These genes were then mapped from EggNOG to the Kyoto Encyclopedia of Genes and Genomes (KEGG) orthology database (http://www.genome.jp/kegg/kegg1.html) using MOCAT2 lookup tables (http://mocat.embl.de/). The annotation of GMMs was performed in R applying Omixer-RPM (http://www.raeslab.org/software/gmms.html). The GMM counts are referring to GMM QMPs based on MGS QMP counts. The GMM abundance table was then transformed using the central log-ratio (CLR) to ensure normality and assess its compositionality nature.

#### 4.2.5 Computation of the phageome from sequenced faecal DNA

Bulk sequence reads reads derived from sequencing of faecal DNA were aligned against Gut Phageome Database (GPD) (46) with BWA mem. Obtained phages were then quality-filtered, retaining only the reads aligning to, at least, 75% of the phage genome length. Phageome counts dataset was then rarefied to the minimal reads and those phages not found in, at least, 10% of the samples (n=21) were removed. This resulted in a total of 502 phages to be included in the final dataset. Phageome counts were then transformed with CLR approach to assess for compositionality of the data.

#### 4.2.6 Computation of differential microbiome features

For the taxonomical analyses, the absolute MGS and/or genus clustered counts were used, after transformation with faecal cell counts. We used linear mixed models, adjusting for age, sex, race, BMI, and dietary data to compute the significance. Multiple testing correction as computed using the False Discovery Rate (FDR) approach. Effect sizes for the differences observe were computed using the Cliff’s Delta test. For the GMM differential analyses, we used the same approach as for the MGSs but using the abundances computations.

### 4.3 Analyses of the untargeted plasma polar metabolites and lipids

#### 4.3.1 Polar metabolites

The plasma samples were stored at −80 °C until analysis. The polar metabolites were analyzed using two-dimensional gas chromatography combined with time-of-flight mass spectrometry (GC×GC-TOFMS, a LECO Pegasus 4D equipped with a consumable-free thermal modulator from LECO Corp). The method has previously been described in detail (36, 43, 47). Specifically, 400 μl methanol and 10 μl internal standard mixture (Heptadecanoic acid-d33, Valine-d8, Glutamic acid-d5 and succinic acid-d4) were added to 30 μl of plasma samples. The samples were vortex mixed and centrifuged for 5 min at 10,000 rpm and half of the supernatant was evaporated to dryness. This was followed by two-step derivatization using methoximation and trimethylsilylation by first adding 25 μl methoxamine (45°C, 60 min) and then 25 μl N-trimethylsilyl-N-methyl trifluoroacetamide (45°C, 60 min). Finally, a retention index standard mixture (n-alkanes) and an injection standard (4,4′ -dibromooctafluorobiphenyl), both in 50 µl hexane, were added to the mixture. The calibration consisted of six points for each quantified metabolite.

The columns were as follows: a phenyl methyl deactivated retention gap (1.5 m × 0.53 mm i.d.) was connected to 10 m × 0.18 mm Rtx-5MS (phase thickness 0.18 μm) and to 1.5 m × 0.1 mm BPX-50 (phase thickness 0.1 μm). Helium was used as the carrier gas at a constant pressure mode (40 psig). A 4-s separation time was used in the second dimension. The temperature program was as follows for the first-dimension column: 50°C (2 min), at 7°C/min to 240°C and at 25°C/min to 300°C (3 min).

The second-dimension column temperature was 15°C higher than the corresponding first-dimension column throughout the program.

ChromaTOF 4.72 vendor software (LECO) was used for within-sample data processing, and the Guineu software (43) was used for alignment, normalization, and peak matching across samples. The normalization was performed by correction with internal standards and specific target metabolites were additionally quantified using external calibration curves. Compounds were identified by comparison to in-house and NIST14 (48) library entries.

Of the 398 total polar metabolites, 143 of them were annotated while the remaining 255 were unannotated. Normalized and log-transformed data was firstly adjusted for individual eGFR (accessible at eGFR Calculator | National Kidney Foundation). The obtained data was then used for the subsequent analyses. We used a combined univariate-multivariate approach to identify relevant features for the discrimination of the T1D individuals from the healthy controls as well as the different T1D subgroups stratified on level of albuminuria. To do so, we used Principal Component Analysis (PCA), Partial Least Squares Discriminant Analysis (PLS-DA) and linear mixed models, with sex, age, BMI, and dietary data as fixed effects. Cliff’s Delta effect size was used to determine the difference in metabolite abundance between the two compared groups. All obtained p-values were adjusted for multiple testing with the false discovery (FDR) approach, considering 10% FDR threshold as significant.

We then used the Human Metabolome Database (HMDB) for annotating polar metabolites enabling a functional enrichment analysis with MetaboAnalyst tool (49). To do so, we uploaded a dataset consisting the abundances of normalized, centered, and scaled HMDB-annotated polar metabolites. Then, we selected the metabolic pathway associated metabolite sets to compute the differentially enriched metabolic pathways in study participants.

#### 4.3.2 Plasma lipidomics

The plasma samples were stored at −80 °C until analysis. The Folch procedure (50) was used for sample preparation with minor modifications based on previously published methods at Steno Diabetes Center Copenhagen (13, 51, 52). Briefly, plasma samples were randomized and lipids were extracted from 10 μL plasma using chloroform:methanol (2:1 v/v) following addition of nine different internal standards (stable isotope labelled and non-physiological lipid species). Samples were analyzed in random order in positive electrospray ionization mode using ultra-high-performance liquid chromatography-quadrupole time-of-flight mass spectrometry (UHPLC-Q-TOF-MS) from Agilent Technologies (Santa Clara, CA, USA). The lipidomics data were pre-processed with MZmine2 (53) in which lipids were semi-quantified by normalizing the peak areas to internal standards and corrected for batch effect. Missing values were imputed with the k-nearest neighbour algorithm and all values were log-2-transformed to achieve normal-distributed data.

For the analysis of lipidomics, we used the same approach as outlined above for the polar metabolites. Of the 7,470 total lipids, 476 of them were annotated, while the remaining 6,994 were unannotated. Following adjustment for individual eGFR value, we used weighted gene co-expression network analysis (WGCNA) to cluster all the strongly correlated lipids, in order to reduce the dimensionality of the data. From the original 7,470 lipids we obtained 122 clusters, ranging from 3 lipids to 1,054 lipids. Lipid clusters were annotated considering their lipid content. If all the lipids in the cluster were unannotated, we named the corresponding cluster as “unknown” followed by a number. From here on in lipids cluster analyses, we followed the same approach as described for the polar metabolites.

Individual annotated lipids were used to study the associations between the clinical groups and the lipid specie types with *LipidomeR* R-package, accessible at https://lipidomer.org/.

#### 4.3.3 Metabolite origin assessment

To evaluate whether metabolome features could be related to bacterial metabolism or to the host’s metabolism and/or lifestyle factors, we used Least Absolute Shrinkage and Selection Operator (LASSO) modeling. This approach allowed us to identify whether the abundance of metabolome features (polar metabolites, lipids and/or lipid clusters) was better predicted by bioclinical data, QMP or GMM data and/or lifestyle.

### 4.4 Drug deconfounding of all data sets in the present study

All the datasets used in this project were scrutinized for any drug-associated features. To do so, we used the R package *metadeconfoundR* (available at https://github.com/TillBirkner/metadeconfoundR) (54), with its default parameters.

### 4.5 Data integration

#### 4.5.1 Multi-omics factor analysis

The normalized datasets were used to investigate interactions and potential signatures involving gut microbiome and plasma metabolome profiles in T1D and its subgroups stratified on albuminuria. Biochemistry data was also combined after normalization (log transformation). Finally, we combined QMP taxonomical counts, GMM CLR-transformed dataset, polar metabolites, lipidomics clusters and biochemistry data for the integration step. We used multi-omics factor analysis (MOFA+) (24) with default parameters, to study the interactions between different layers of data and the potential for identifying combinations of features useful for predicting T1D with varying levels of albuminuria.

### 4.6 Statistical analysis

All statistical analyses were performed in R software (https://cran.r-project.org/), running version 4.1.0. Significance tests results were corrected for multiple testing with False Discovery Rate (FDR) approach and significance set at 10% FDR threshold. Data visualization was done with *ggplot2* R package. Specific details on the methodology regarding specific data types has been reported in the corresponding section of the methods.

## Data availability statement

The datasets presented in this study can be found in online repositories. The names of the repository/repositories and accession number(s) can be found in the article/**Supplementary Material**.

## Ethics statement

The studies involving human participants were reviewed and approved by Ethics Committee of the Danish Capital Region (protocol H-15018107). The patients/participants provided their written informed consent to participate in this study.

## Author contributions

Conceptualization: OP, PR, TSA, and SW. Methodology: MC-G, TSA, PH, JV YF, LL, ES, MA, TH and CL-Q. Investigation: MC-G, TSA, SW, YF, JV, LL, ES, PH, and CL-Q. Visualization: MC-G. Funding acquisition: PR. Project administration: PR, OP. Supervision: OP, PR. Writing – original draft: MC-G, TSA. Writing – review & editing: All authors. All authors contributed to the article and approved the submitted version.

## Funding

This work was supported by the Novo Nordisk Foundation (grant number NNF14OC0013659) PROTON Personalising treatment of diabetic kidney disease; internal funding was provided by Steno Diabetes Center Copenhagen (SDCC), Herlev, Denmark. TSA was supported by the Novo Nordisk Foundation (Grant # NNF18OC0052457) and SDCC.

## Acknowledgments

We thank all the study participants and the SDCC laboratory staff for their contributions.

## Conflict of interest

PR reports personal fees from Bayer during the conduct of the study. He has received research support and personal fees from AstraZeneca and Novo Nordisk, and personal fees from Astellas Pharma, Boehringer Ingelheim, Eli Lilly, Gilead Sciences, Mundipharma, Sanofi, and Vifor Pharma. All fees are given to Steno Diabetes Center Copenhagen.

Author MC-G was employed by company LEITAT Technological Center. Author JKV was employed by Clinical Microbiomics.

The remaining authors declare that the research was conducted in the absence of any commercial or financial relationships that could be construed as a potential conflict of interest.

## Publisher’s note

All claims expressed in this article are solely those of the authors and do not necessarily represent those of their affiliated organizations, or those of the publisher, the editors and the reviewers. Any product that may be evaluated in this article, or claim that may be made by its manufacturer, is not guaranteed or endorsed by the publisher.

## References

[1] Saran R Robinson B Abbott KC Agodoa LYC Bragg-Gresham J Balkrishnan R. US Renal data system 2018 annual data report: Epidemiology of kidney disease in the united states. Am J Kidney Dis (2019) 73:A7–8. doi: 10.1053/j.ajkd.2019.01.001 PMC6620109 30798791

[2] Bikbov B Purcell CA Levey AS Smith M Abdoli A Abebe M. Global, regional, and national burden of chronic kidney disease, 1990–2017: a systematic analysis for the global burden of disease study 2017. Lancet (2020) 395:709–33. doi: 10.1016/S0140-6736(20)30045-3 PMC7049905 32061315

[3] Carrero JJ Grams ME Sang Y Ärnlöv J Gasparini A Matsushita K. Albuminuria changes are associated with subsequent risk of end-stage renal disease and mortality. Kidney Int (2017) 91:244–51. doi: 10.1016/j.kint.2016.09.037 PMC5523054 27927597

[4] Bourlioux P Koletzko B Guarner F Braesco Véronique. The intestine and its microflora are partners for the protection ofthe host: report on the danone symposium “The IntelligentIntestine,” held in Paris, June 14, 2002. Am J Clin Nutr (2003) 78:675–83. doi: 10.1093/ajcn/78.4.675 14522724

[5] Vaarala O Atkinson MA Neu J. The “perfect storm” for type 1 diabetes: The complex interplay between intestinal microbiota, gut permeability, and mucosal immunity. Diabetes (2008) 57:2555–62. doi: 10.2337/db08-0331 PMC2551660 18820210

[6] Anders HJ Andersen K Stecher B. The intestinal microbiota, a leaky gut, and abnormal immunity in kidney disease. Kidney Int (2013) 83:1010–6. doi: 10.1038/ki.2012.440 23325079

[7] le Chatelier E Nielsen T Qin J Prifti E Hildebrand F Falony G. Richness of human gut microbiome correlates with metabolic markers. Nature (2013) 500:541–6. doi: 10.1038/nature12506 23985870

[8] Zheng P Li Z Zhou Z. Gut microbiome in type 1 diabetes: A comprehensive review. Diabetes Metab Res Rev (2018) 34:1–9. doi: 10.1002/dmrr.3043 PMC6220847 29929213

[9] Arneth B Arneth R Shams M. Metabolomics of type 1 and type 2 diabetes. Int J Mol Sci (2019) 20:1–14. doi: 10.3390/ijms20102467 PMC6566263 31109071

[10] Lu J Chen PP Zhang JX Li XQ Wang GH Yuan BY. GPR43 deficiency protects against podocyte insulin resistance in diabetic nephropathy through the restoration of AMPKα activity. Theranostics (2021) 11:4728–42. doi: 10.7150/thno.56598 PMC7978296 33754024

[11] Kikuchi K Saigusa D Kanemitsu Y Matsumoto Y Thanai P Suzuki N. Gut microbiome-derived phenyl sulfate contributes to albuminuria in diabetic kidney disease. Nat Commun (2019) 10 :1835. doi: 10.1038/s41467-019-09735-4 31015435 PMC6478834

[12] Winther SA Henriksen P Vogt JK Hansen TH Ahonen L Suvitaival T. Gut microbiota profile and selected plasma metabolites in type 1 diabetes without and with stratification by albuminuria. Diabetologia (2020) 63:2713–24. doi: 10.1007/s00125-020-05260-y 32886190

[13] Tofte N Suvitaival T Ahonen L Winther SA Theilade S Frimodt-Møller M. Lipidomic analysis reveals sphingomyelin and phosphatidylcholine species associated with renal impairment and all-cause mortality in type 1 diabetes. Sci Rep (2019) 9:1–10. doi: 10.1038/s41598-019-52916-w 31705008 PMC6841673

[14] Mäkinen VP Tynkkynen T Soininen P Forsblom C Peltola T Kangas AJ. Sphingomyelin is associated with kidney disease in type 1 diabetes (The FinnDiane study). Metabolomics (2012) 8:369–75. doi: 10.1007/s11306-011-0343-y PMC3351624 22661917

[15] Klein RL Hammad SM Baker NL Hunt KJ al Gadban MM Cleary PA. Decreased plasma levels of select very long chain ceramide species are associated with the development of nephropathy in type 1 diabetes. Metabolism (2014) 63:1287–95. doi: 10.1016/j.metabol.2014.07.001 PMC5894336 25088746

[16] Mäkinen VP Tynkkynen T Soininen P Peltola T Kangas AJ Forsblom C. Metabolic diversity of progressive kidney disease in 325 patients with type 1 diabetes (the FinnDiane study). J Proteome Res (2012) 11:1782–90. doi: 10.1021/pr201036j 22204613

[17] Kim JK Shin S-Y Moon JS Li L Cho SK Kim T-J. Isolation of dextran-hydrolyzing intestinal bacteria and characterization of their dextranolytic activities. Biopolymers (2015) 103:321–7. doi: 10.1002/bip.22615 25652688

[18] Vieira-Silva S Falony G Darzi Y Lima-Mendez G Garcia Yunta R Okuda S. Species-function relationships shape ecological properties of the human gut microbiome. Nat Microbiol (2016) 1 :16088. doi: 10.1038/nmicrobiol.2016.88 27573110

[19] Argelaguet R Arnol D Bredikhin D Deloro Y Velten B Marioni JC. MOFA+: A statistical framework for comprehensive integration of multi-modal single-cell data. Genome Biol (2020) 21:1–17. doi: 10.1186/s13059-020-02015-1 PMC7212577 32393329

[20] Durack J v. Lynch S. The gut microbiome: Relationships with disease and opportunities for therapy. J Exp Med (2019) 216:20–40. doi: 10.1084/jem.20180448 30322864 PMC6314516

[21] Parada Venegas D de la Fuente MK Landskron G González MJ Quera R Dijkstra G. Corrigendum: Short chain fatty acids (SCFAs)-mediated gut epithelial and immune regulation and its relevance for inflammatory bowel diseases. Front Immunol (2019) 10:1486. doi: 10.3389/fimmu.2019.01486 31316522 PMC6611342

[22] Qin Q Yan S Yang Y Chen J Li T Gao X. A metagenome-wide association study of the gut microbiome and metabolic syndrome. Front Microbiol (2021) 12:682721. doi: 10.3389/fmicb.2021.682721 34335505 PMC8322780

[23] Bell A Juge N. Mucosal glycan degradation of the host by the gut microbiota. Glycobiology (2021) 31:691–6. doi: 10.1093/glycob/cwaa097 PMC8252862 33043970

[24] Yao Y Yan L Chen H Wu N Wang W Wang D. Cyclocarya paliurus polysaccharides alleviate type 2 diabetic symptoms by modulating gut microbiota and short-chain fatty acids. Phytomedicine (2020) 77:153268. doi: 10.1016/j.phymed.2020.153268 32663709

[25] Shkoporov AN v. Chaplin A Shcherbakova VA Suzina NE Kafarskaia LI Bozhenko VK. Ruthenibacterium lactatiformans gen. nov., sp. nov., an anaerobic, lactate-producing member of the family ruminococcaceae isolated from human faeces. Int J Syst Evol Microbiol (2016) 66:3041–9. doi: 10.1099/ijsem.0.001143 27154556

[26] Brouwers MCGJ Ham JC Wisse E Misra S Landewe S Rosenthal M. Elevated lactate levels in patients with poorly regulated type 1 diabetes and glycogenic hepatopathy: A new feature of mauriac syndrome. Diabetes Care (2015) 38:e11–2. doi: 10.2337/dc14-2205 25614691

[27] Li H Xu H Li Y Jiang Y Hu Y Liu T. Alterations of gut microbiota contribute to the progression of unruptured intracranial aneurysms. Nat Commun (2020) 11:3218. doi: 10.1038/s41467-020-16990-3 PMC7316982 32587239

[28] Kim KS Oh DH Kim JY Lee BG You JS Chang KJ. Taurine ameliorates hyperglycemia and dyslipidemia by reducing insulin resistance and leptin level in otsuka long-Evans tokushima fatty (OLETF) rats with long-term diabetes. Exp Mol Med (2012) 44:665–73. doi: 10.3858/emm.2012.44.11.075 PMC3509183 23114424

[29] Ito T Schaffer SW Azuma J. The potential usefulness of taurine on diabetes mellitus and its complications. Amino Acids (2012) 42:1529–39. doi: 10.1007/s00726-011-0883-5 PMC3325402 21437784

[30] Johnson EL Heaver SL Waters JL Kim BI Bretin A Goodman AL. Sphingolipids produced by gut bacteria enter host metabolic pathways impacting ceramide levels. Nat Commun (2020) 11:1–11. doi: 10.1038/s41467-020-16274-w 32424203 PMC7235224

[31] Chaurasia B Tippetts TS Monibas RM Liu J Li Y Wang L. Targeting a ceramide double bond improves insulin resistance and hepatic steatosis. Science (2019) 365:386–92. doi: 10.1126/science.aav3722 PMC6787918 31273070

[32] Xie C Jiang C Shi J Gao X Sun D Sun L. An intestinal farnesoid x receptor-ceramide signaling axis modulates hepatic gluconeogenesis in mice. Diabetes (2017) 66:613–26. doi: 10.2337/db16-0663 PMC5319721 28223344

[33] Cohen LJ Esterhazy D Kim SH Lemetre C Aguilar RR Gordon EA. Commensal bacteria make GPCR ligands that mimic human signalling molecules. Nature (2017) 549:48–53. doi: 10.1038/nature23874 28854168 PMC5777231

[34] Heaver SL Johnson EL Ley RE. Sphingolipids in host–microbial interactions. Curr Opin Microbiol (2018) 43:92–9. doi: 10.1016/j.mib.2017.12.011 29328957

[35] Karaki SI Mitsui R Hayashi H Kato I Sugiya H Iwanaga T. Short-chain fatty acid receptor, GPR43, is expressed by enteroendocrine cells and mucosal mast cells in rat intestine. Cell Tissue Res (2006) 324:353–60. doi: 10.1007/s00441-005-0140-x 16453106

[36] Tofte N Suvitaival T Trost K Mattila IM Theilade S Winther SA. Metabolomic assessment reveals alteration in polyols and branched chain amino acids associated with present and future renal impairment in a discovery cohort of 637 persons with type 1 diabetes. Front Endocrinol (Lausanne) (2019) 10:818. doi: 10.3389/fendo.2019.00818 31824430 PMC6883958

[37] Hansen CS Suvitaival T Theilade S Mattila I Lajer M Trošt K. Cardiovascular autonomic neuropathy in type 1 diabetes is associated with disturbances in TCA, lipid, and gucose metabolism. Front Endocrino (2022) 13, 831793. doi: 10.3389/fendo.2022.831793 PMC9046722 35498422

[38] Maier L Pruteanu M Kuhn M Zeller G Telzerow A Anderson EE. Extensive impact of non-antibiotic drugs on human gut bacteria. Nature (2018) 555:623–8. doi: 10.1038/nature25979 PMC6108420 29555994

[39] Zimmermann M Zimmermann-Kogadeeva M Wegmann R Goodman AL. Mapping human microbiome drug metabolism by gut bacteria and their genes. Nature (2019) 570:462–7. doi: 10.1038/s41586-019-1291-3 PMC6597290 31158845

[40] Hiltunen TP Rimpelä JM Mohney RP Stirdivant SM Kontula KK. Effects of four different antihypertensive drugs on plasma metabolomic profiles in patients with essential hypertension. PloS One (2017) 12:1–16. doi: 10.1371/journal.pone.0187729 PMC5679533 29121091

[41] Leshem A Segal E Elinav E. The gut microbiome and individual-specific responses to diet. mSystems (2020) 5:1–12. doi: 10.1128/mSystems.00665-20 PMC7527138 32994289

[42] Mailing LJ Allen JM Buford TW Fields CJ Woods JA. Exercise and the gut microbiome: A review of the evidence, potential mechanisms, and implications for human health. Exerc Sport Sci Rev (2019) 47:75–85. doi: 10.1249/JES.0000000000000183 30883471

[43] Pedersen HK Gudmundsdottir V Nielsen HB Hyotylainen T Nielsen T Chatelier L. Human gut microbes impact host serum metabolome and insulin sensitivity. Nature (2016) 535:376–81. doi: 10.1038/nature18646 27409811

[44] Nielsen HB Almeida M Juncker AS Rasmussen S Li J Sunagawa S. Identification and assembly of genomes and genetic elements in complex metagenomic samples without using reference genomes. Nat Biotechnol (2014) 32:822–8. doi: 10.1038/nbt.2939 24997787

[45] Vandeputte D Kathagen G D’Hoe K Vieira-Silva S Valles-Colomer M Sabino J. Quantitative microbiome profiling links gut community variation to microbial load. Nature (2017) 551:507–11. doi: 10.1038/nature24460 29143816

[46] Camarillo-Guerrero LF Almeida A Rangel-Pineros G Finn RD Lawley TD. Massive expansion of human gut bacteriophage diversity. Cell (2021) 184:1098–1109.e9. doi: 10.1016/j.cell.2021.01.029 33606979 PMC7895897

[47] Castillo S Mattila I Miettinen J Orešič M Hyötyläinen T. Data analysis tool for comprehensive two-dimensional gas chromatography/time-of-flight mass spectrometry. Anal Chem (2011) 83:3058–67. doi: 10.1021/ac103308x 21434611

[48] Bowden JA Heckert A Ulmer CZ Jones CM Koelmel JP Abdullah L. Harmonizing lipidomics: NIST interlaboratory comparison exercise for lipidomics using SRM 1950-metabolites in frozen human plasma. J Lipid Res (2017) 58:2275–88. doi: 10.1194/jlr.M079012 PMC5711491 28986437

[49] Pang Z Chong J Zhou G de Lima Morais DA Chang L Barrette M. MetaboAnalyst 5.0: Narrowing the gap between raw spectra and functional insights. Nucleic Acids Res (2021) 49:W388–96. doi: 10.1093/nar/gkab382 PMC8265181 34019663

[50] Folch J Lees M Stanley GHS. A simple method for the isolation and purification of total lipides from animal tissues. J Biol Chem (1957) 226:497–509. doi: 10.1016/S0021-9258(18)64849-5 13428781

[51] Luukkonen PK Zhou Y Nidhina Haridas PA Dwivedi OP Hyötyläinen T Ali A. Impaired hepatic lipid synthesis from polyunsaturated fatty acids in TM6SF2 E167K variant carriers with NAFLD. J Hepatol (2017) 67:128–36. doi: 10.1016/j.jhep.2017.02.014 28235613

[52] O’Gorman A Suvitaival T Ahonen L Cannon M Zammit S Lewis G. Identification of a plasma signature of psychotic disorder in children and adolescents from the Avon longitudinal study of parents and children (ALSPAC) cohort. Transl Psychiatry (2017) 7:e1240. doi: 10.1038/tp.2017.211 28949339 PMC5639252

[53] Pluskal T Castillo S Villar-Briones A Orešič M. MZmine 2: Modular framework for processing, visualizing, and analyzing mass spectrometry-based molecular profile data. BMC Bioinf (2010) 11 :395. doi: 10.1186/1471-2105-11-395 PMC2918584 20650010

[54] Forslund SK Chakaroun R Zimmermann-Kogadeeva M Markó L Aron-Wisnewsky J Nielsen T. Combinatorial, additive and dose-dependent drug–microbiome associations. Nature (2021) 600:500–5. doi: 10.1038/s41586-021-04177-9 34880489

