# Supplementary material for: Multiomics signatures of type 1 diabetes with and without albuminuria
Source: Front Endocrinol (Lausanne). 2022 Dec 2;13:1015557. doi: 10.3389/fendo.2022.1015557 (PMC9755599; doi:10.3389/fendo.2022.1015557)
Supplement: Supplementary file 1 [file DataSheet_1.docx]

## **Supplementary data**

| **Table of Contents** | **Page** |
| --- | --- |
| **Supplementary Figures** |  |
| Supplementary figure 1 | …………………………………………………. 5 |
| Supplementary figure 2 | …………………………………………………. 5 |
| Supplementary figure 3 | …………………………………………………. 6 |
| Supplementary figure 4 | …………………………………………………. 6 |
| Supplementary figure 5 | …………………………………………………. 7 |
| Supplementary figure 6 | …………………………………………………. 7 |
| Supplementary figure 7 | …………………………………………………. 8 |
| Supplementary figure 8 | …………………………………………………. 8 |
| Supplementary figure 9 | …………………………………………………. 9 |
| Supplementary figure 10 | …………………………………………………. 9 |
| Supplementary figure 11 | …………………………………………………. 10 |
| Supplementary figure 12 | …………………………………………………. 10 |
| Supplementary figure 13 | …………………………………………………. 11 |
| Supplementary References | …………………………………………………. 12 |

**Supplementary Figure Legends**

**Supplementary Figure 1:** **Gut bacterial gene richness distribution among participating (albuminuria stratified type 1 diabetes and healthy control) groups.** Violin plots combined with points show the distribution of the bacterial gene richness per sample group (x-axis), while the boxplots inside indicate the median value of the sample group distribution with a horizontal bar and the upper and lower limits of the distribution with vertical bars. Significance assessment is indicated per each diversity index as Kruskal-Wallis p-value for the multigroup comparison and with horizontal bars with the corresponding p-value for the pairwise comparisons. KO = KEGG Orthology

**Supplementary Figure 2:** **Alpha diversity indexes for the participating (albuminuria stratified type 1 diabetes and healthy control) groups**. For each index, the distribution of the alpha diversity indexes is displayed in a form of a combined violin and point plot. The summary statistics of these distributions are indicated as a boxplot, with the median of the distribution indicated with a horizontal bar and the upper and lower limits of the distribution with vertical bars. Significance assessment is indicated per each diversity index as Kruskal-Wallis p-value for the multigroup comparison and with horizontal bars with the corresponding p-value for the pairwise comparisons.

**Supplementary Figure 3:** **Taxonomical composition of the participating study groups.** Barplots represent the taxonomical composition of the samples metagenomic communities, clustered at genus level and divided by each participating (albuminuria stratified and healthy control) group. Genus composition has been computed from the quantitative microbial profile (QMP) counts and, for visualization purposes, all entries have been divided by 108. Top 15 genera have been individually colored, while the remaining ones are depicted as “Other”.

**Supplementary Figure 4:** **Principal Coordinates Analysis (PCoA) of the metagenomic communities for the 4 participating study groups, based on the Jensen-Shannon Divergence index’s distance matrix**. Ellipses have been drawn indicating the multidimensional space occupied for each of the groups. Distribution of the participants in each of the principal coordinates (PC1 and PC2) on x and y axis, are also indicated as density plots (PC1 in the top-horizontal axis and PC2 on the right-vertical axis).

**Supplementary Figure 5:** **Taxonomical differences between T1D with micro-albuminuria and healthy controls at metagenomic species (MGS) level.** In the left panel, A volcano plot showing difference in absolute abundance of metagenomic species (MGS) between healthy controls (n=50) and T1D individuals with micro-albuminuria (n=50). X-axis indicates Cliff’s Delta effect size; Y-axis represents FDR-corrected (negative log) p–values. MGS that associated positively with T1D micro-albuminuria group have been depicted towards increasing direction of effects (right). MGS circle color depicts the corresponding annotated phylum. Circle size corresponds to the number of individuals in the cohort where the specific MGS was found. Transparency of the circle corresponds to the average relative abundance in which each MGS is found within participants.

In the right panel, the Cliff’s Delta effect sizes for the significantly differing MGS are depicted as horizontal bars: green for higher and red for lower MGS abundances within T1D micro-albuminuria individuals.

**Supplementary Figure 6:** **Taxonomical differences between T1D with macro-albuminuria and healthy controls at metagenomic species (MGS) level.** In the left panel, A volcano plot showing difference in absolute abundance of metagenomic species (MGS) between healthy controls (n=50) and T1D individuals with macro-albuminuria (n=50). X-axis indicates Cliff’s Delta effect size; Y-axis represents FDR-corrected (negative log) p–values. MGS that associated positively with T1D macro-albuminuria group have been depicted towards increasing direction of effects (right). MGS circle color depicts the corresponding annotated phylum. Circle size corresponds to the number of individuals in the cohort where the specific MGS was found. Transparency of the circle corresponds to the average relative abundance in which each MGS is found within participants.

In the right panel, the Cliff’s Delta effect sizes for the significantly differing MGS are depicted as horizontal bars: green for higher and red for lower MGS abundances within T1D macro-albuminuria individuals.

**Supplementary Figure 7:** **MGS differences between T1D subgroups compared to healthy controls.** **A:** ternary plot for the MGS relative abundance dataset in the T1D cohort. Each triangle vertex represents the maximum relative abundance in one of the three T1D subgroups: normo-, micro- and macro-albuminuria. The points represent each of the MGS, colored by phylum and the size of the points are relative to the total abundance of the MGS in the cohort. **B:** Venn diagram showing the shared and unique differential MGS between macro-albuminuria and healthy individuals (in red) and between the micro-albuminuria and healthy individuals (in blue).

**Supplementary Figure 8:** Differential Gut Metabolic Modules (GMM) enriched between healthy control and T1D groups. The left panel shows the volcano plot where significantly enriched GMMs (FDR < 10%) between the two groups have been colored in red and the five most significant GMMs annotated. In the right panel: the Cliff’s Delta effect sizes for the significantly differing MGS are depicted as horizontal bars: green for decreased and red for increased GMM abundances within T1D individuals compared to HCs.

**Supplementary Figure 9: Associations between phageome composition and clinical characteristics.** Associations are based on linear regression models (corrected for age, sex, and diet) with significant findings (FDR<10%) presented in this figure. Directionality of effects is indicated by Cliff’s delta effect size in a blue-to-red gradient, blue denoting a negative, while red denoting a positive association. Phages are presented on the x-axis with their annotation ID while the bio-clinical factors are listed on the y-axis.

**Supplementary Figure 10:** PLS-DA approach results for the comparison between macro- and micro-albuminuria T1D individuals. A: PLS-DA model summary statistics. As can be seen on the top-left panel, while the explained variance of the model is good for the training model (R2) the reproducibility low (Q2), indicating strong overfitting. B: PCA plot of the polar metabolites filtered dataset for the most contributing factors. As shown, no separation was achieved between the two compared groups.

**Supplementary Figure 11:** **Univariate analysis of the polar metabolites between micro- and macro-albuminuria individuals with T1D.** Volcano plot showing the Cliff’s Delta effect size in the horizontal axis and the FDR adjusted p-value in the vertical axis. Significant metabolites (FDR<10%) have been colored in red and labeled with the corresponding annotation. For unannotated metabolites a random number was assigned.

**Supplementary Figure 12:** Top contributors for the selected factors (2 and 3) in the multi-omics factor analysis. For both factors 2 (top panel) and 3 (bottom panel), biochemical (left) and polar metabolites (right) are displayed. Directionality of the selected features is indicated with positive (+) and negative (-) symbols, depending on whether they are directly related (+) or inversely (-) related to T1D. x-axis displays the contributing variables while y-axis represents %weight contributed.

**Supplementary Figure 13: Metabolite origin assessment.** From left to right, metabolite origins for the polar metabolites, complete lipidomics dataset and lipid clusters. For each dataset, we considered 4 potential origins: host biochemical related factors, microbiome origin as assessed by bacterial abundances (QMP) and bacterial functional profiles (GMM) and lifestyle and dietary factors. In each panel, a set of points represent each metabolite that was found to be partially explained by some of the potential origin features. A combination of violin and boxplot represent the range of explained metabolite abundance for each data type. The percentage of the metabolites explained are indicated under the violin plot for each data type.

**Supplementary Figures**

**Supplementary Figure 1**


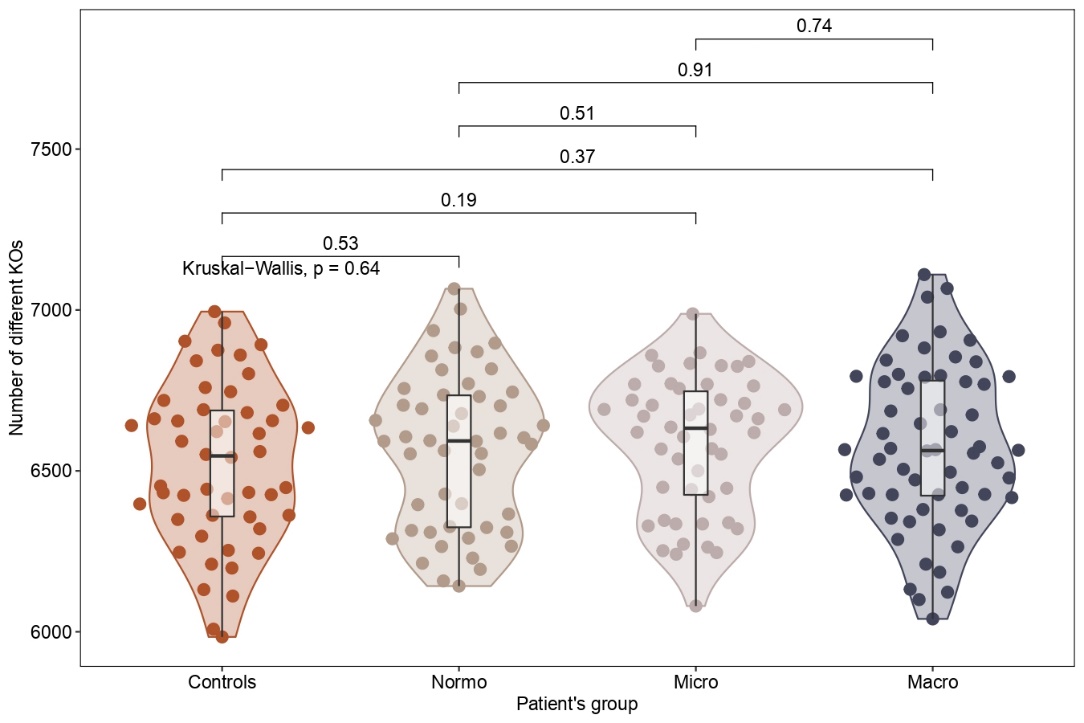


**Supplementary Figure 2**


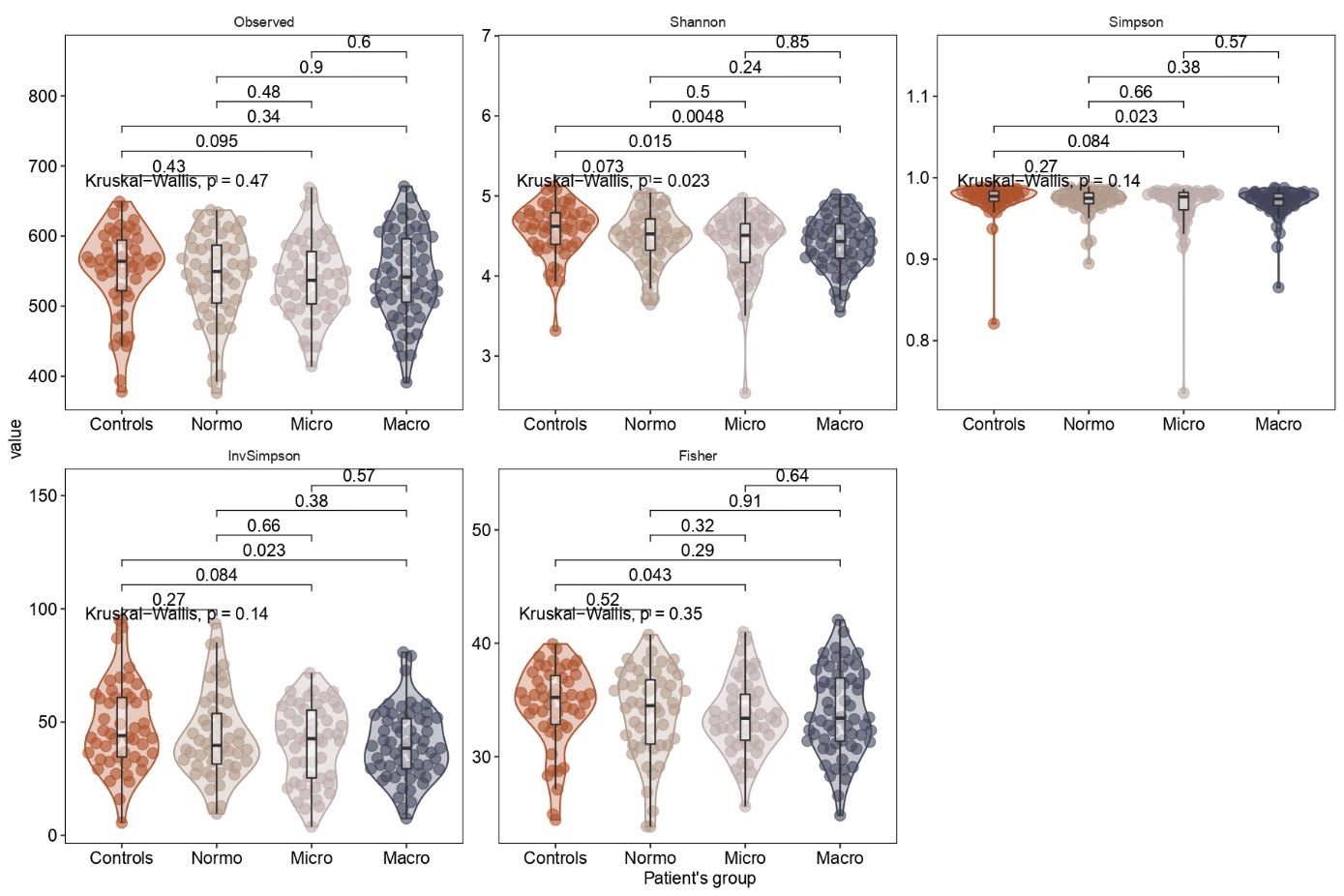


**Supplementary Figure 3**


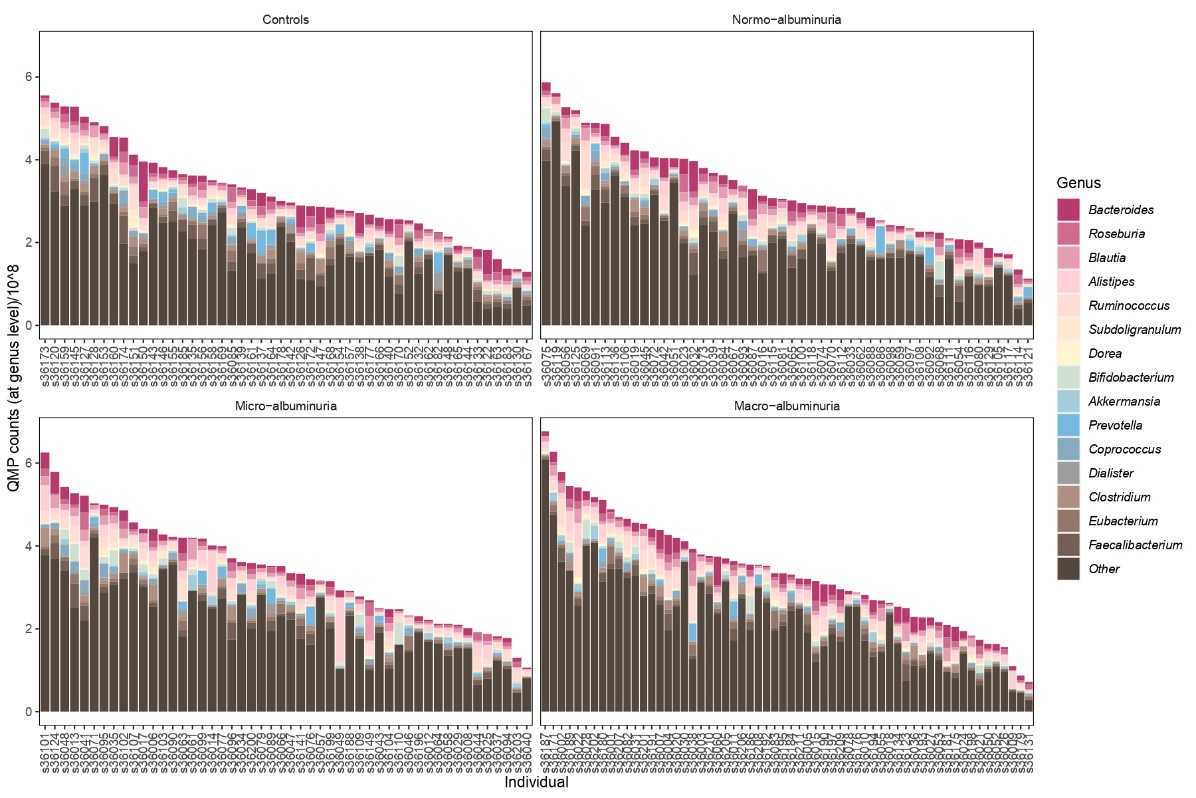


**Supplementary Figure 4**


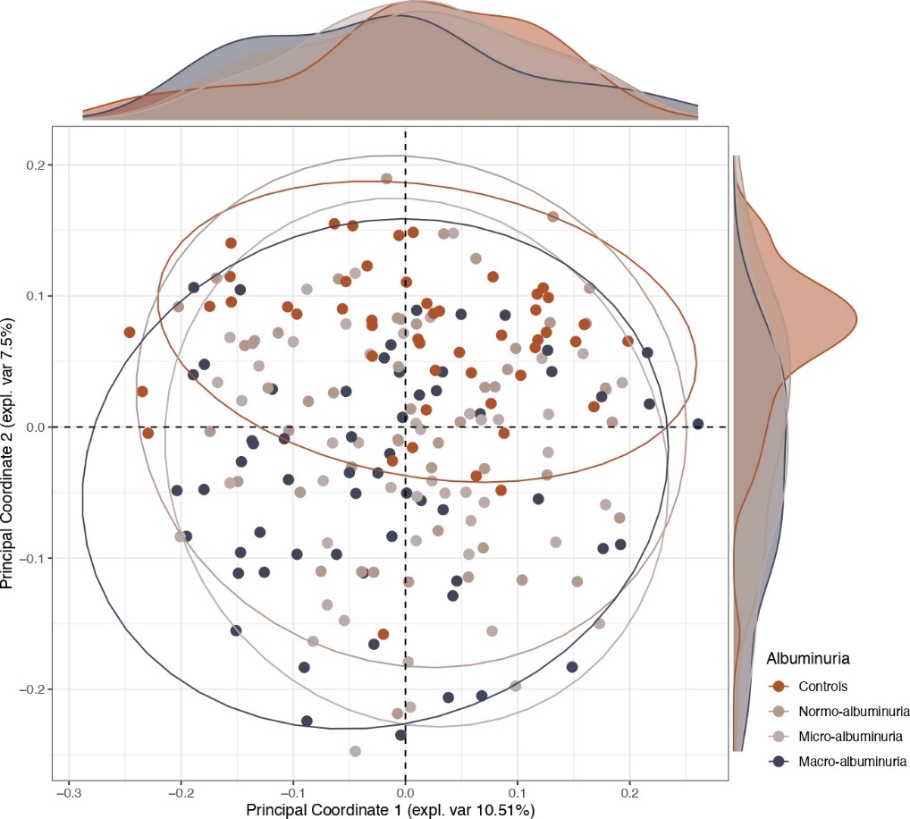


**Supplementary Figure 5**


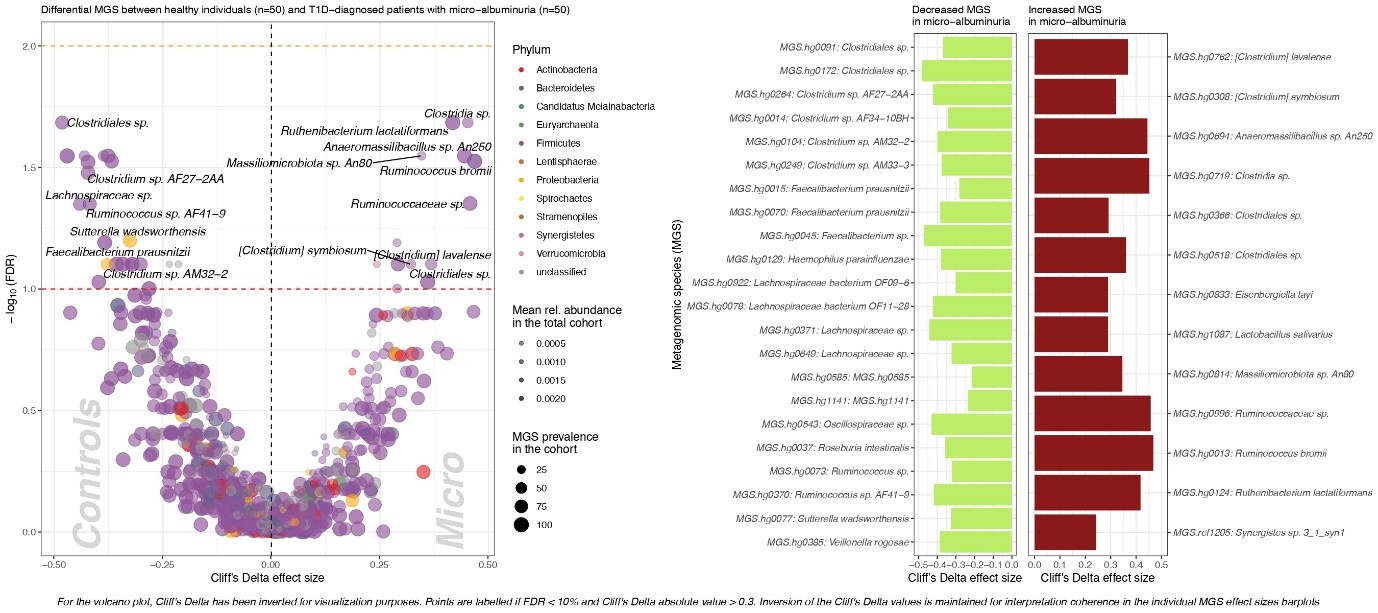


**Supplementary Figure 6**
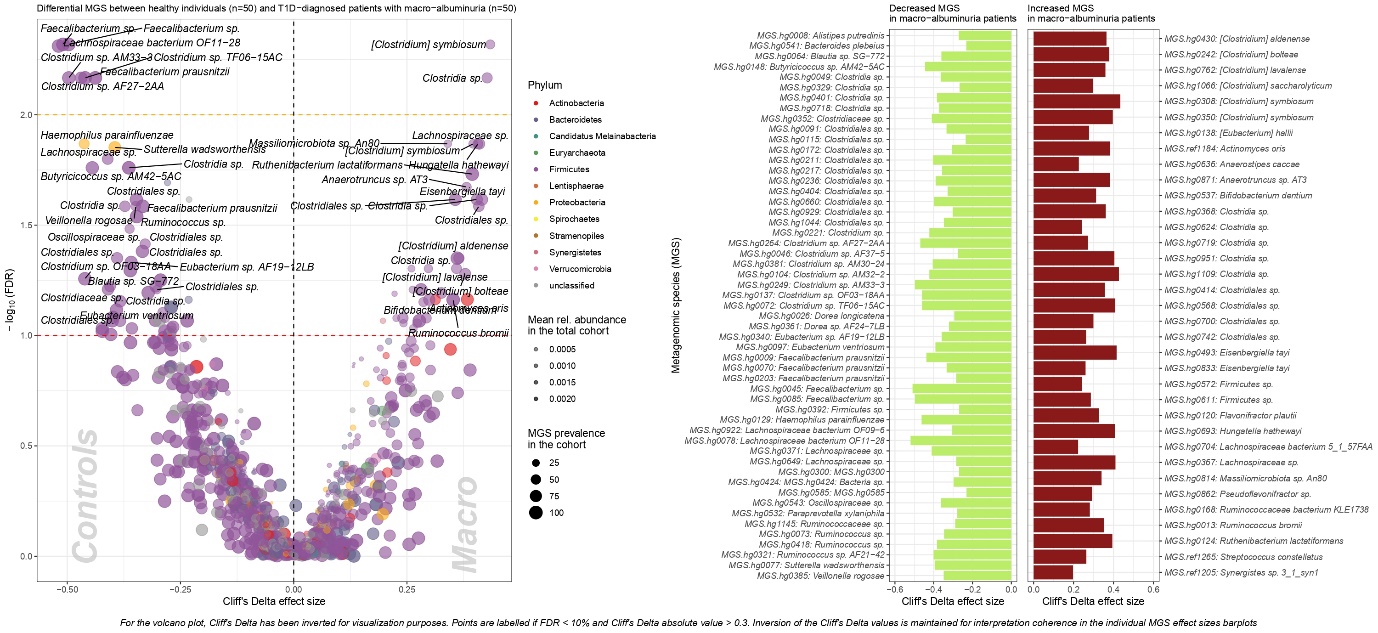


**Supplementary Figure 7**


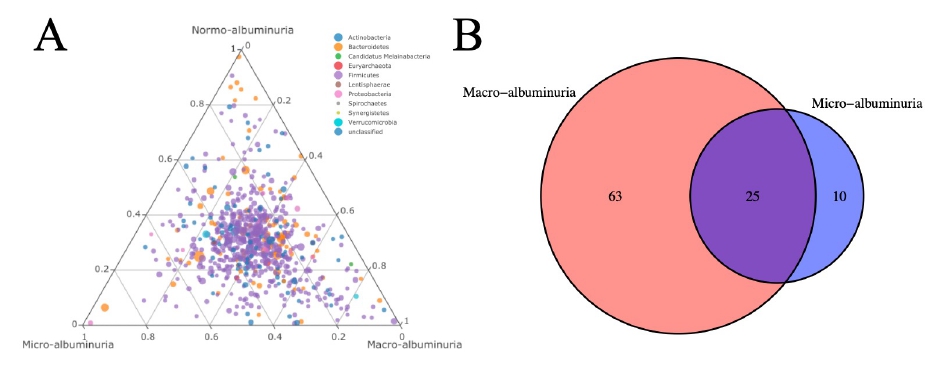


**Supplementary Figure 8**


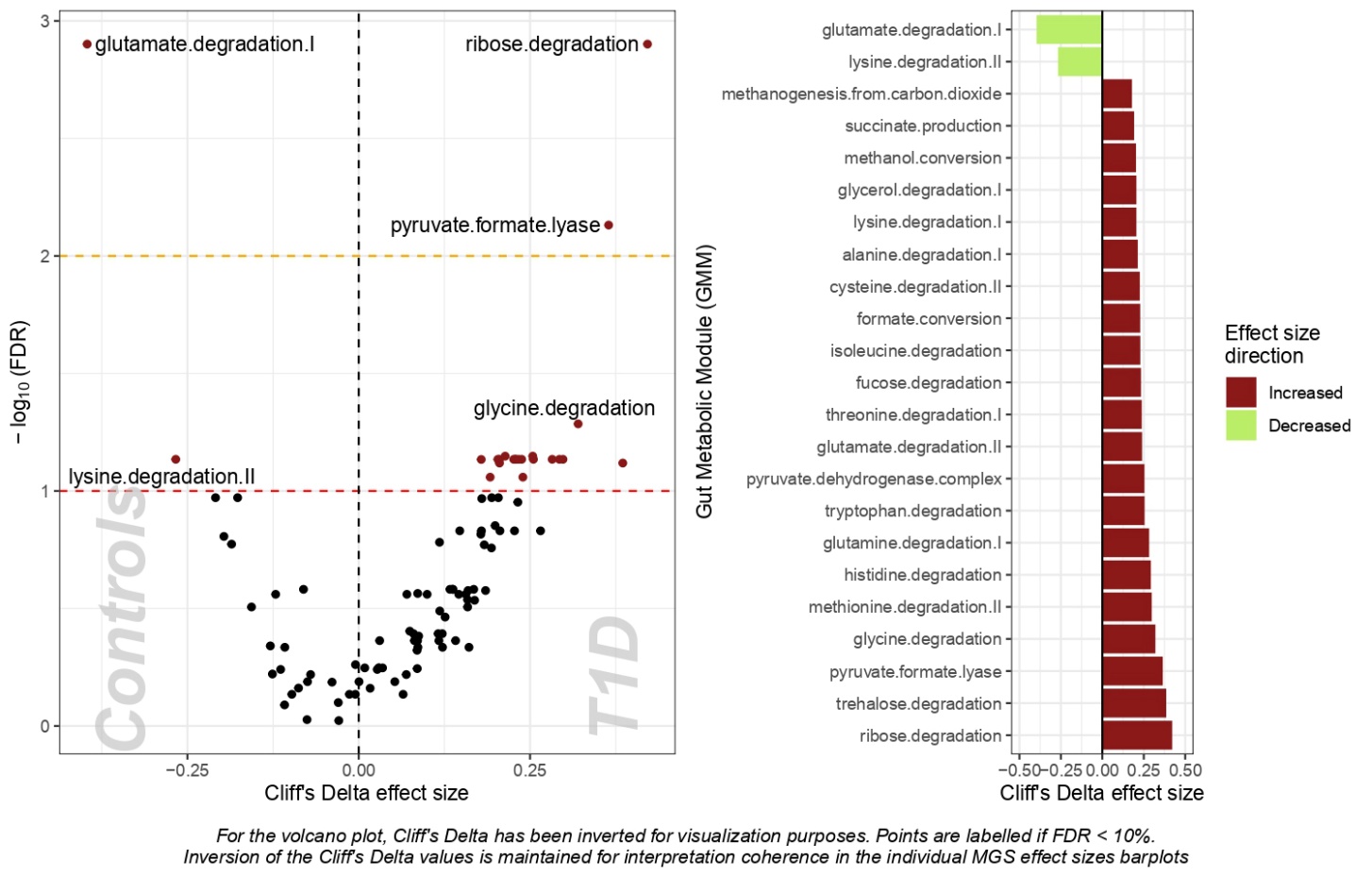


**Supplementary Figure 9**


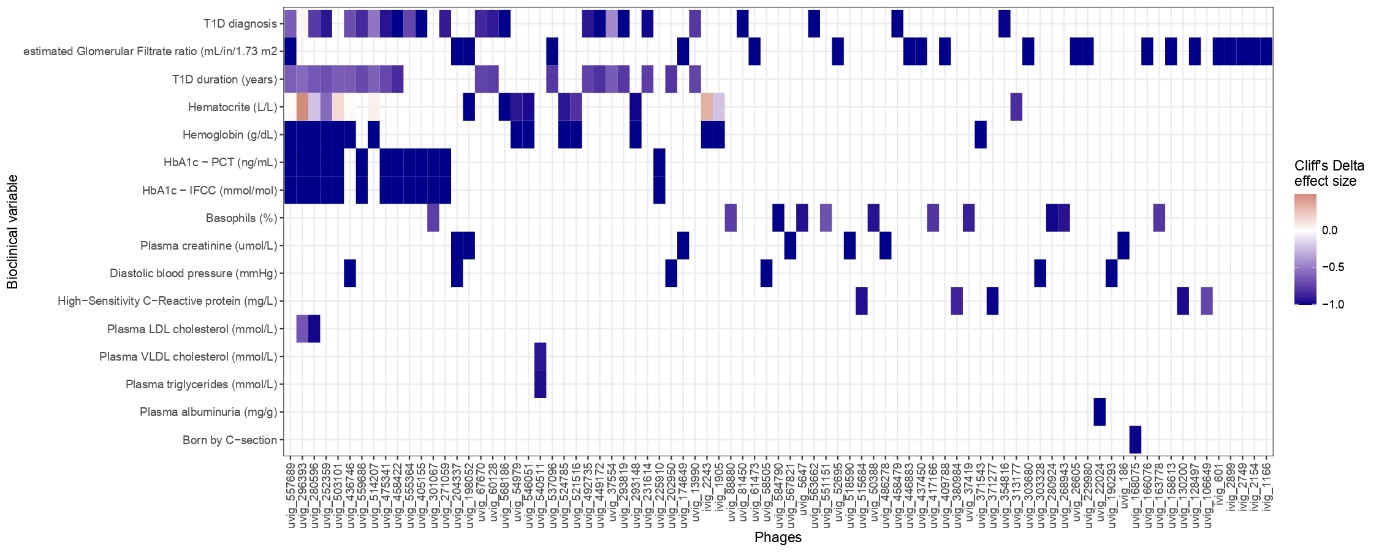


**Supplementary Figure 10**


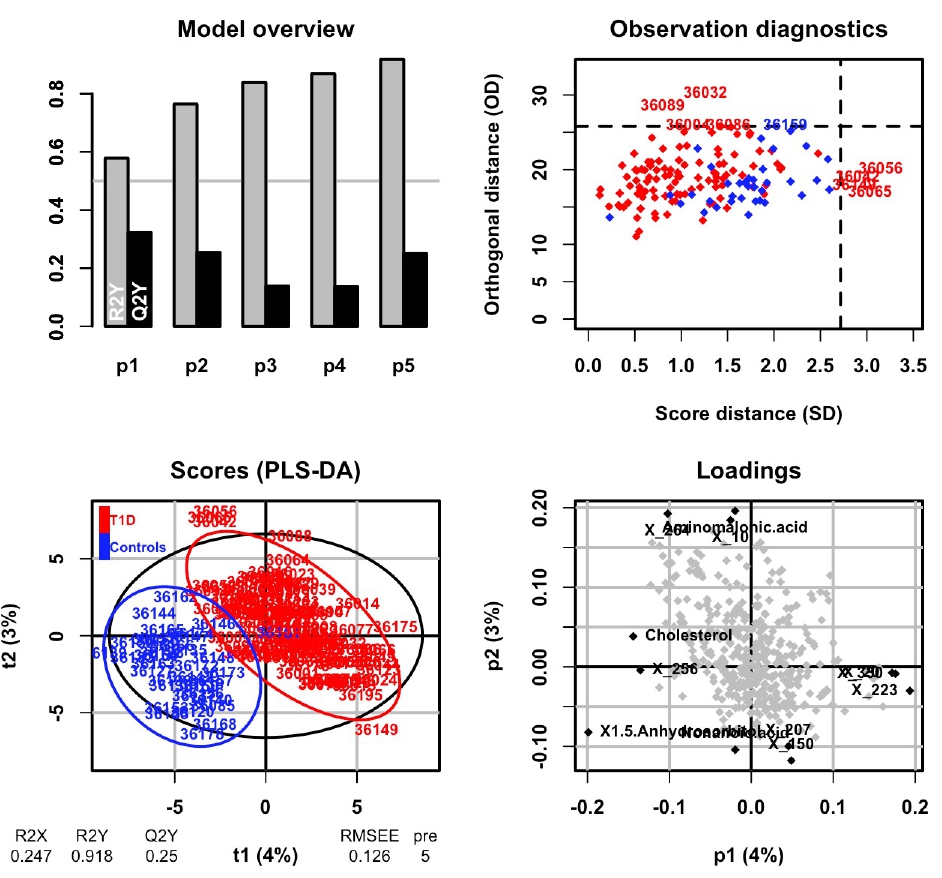


**Supplementary Figure 11**


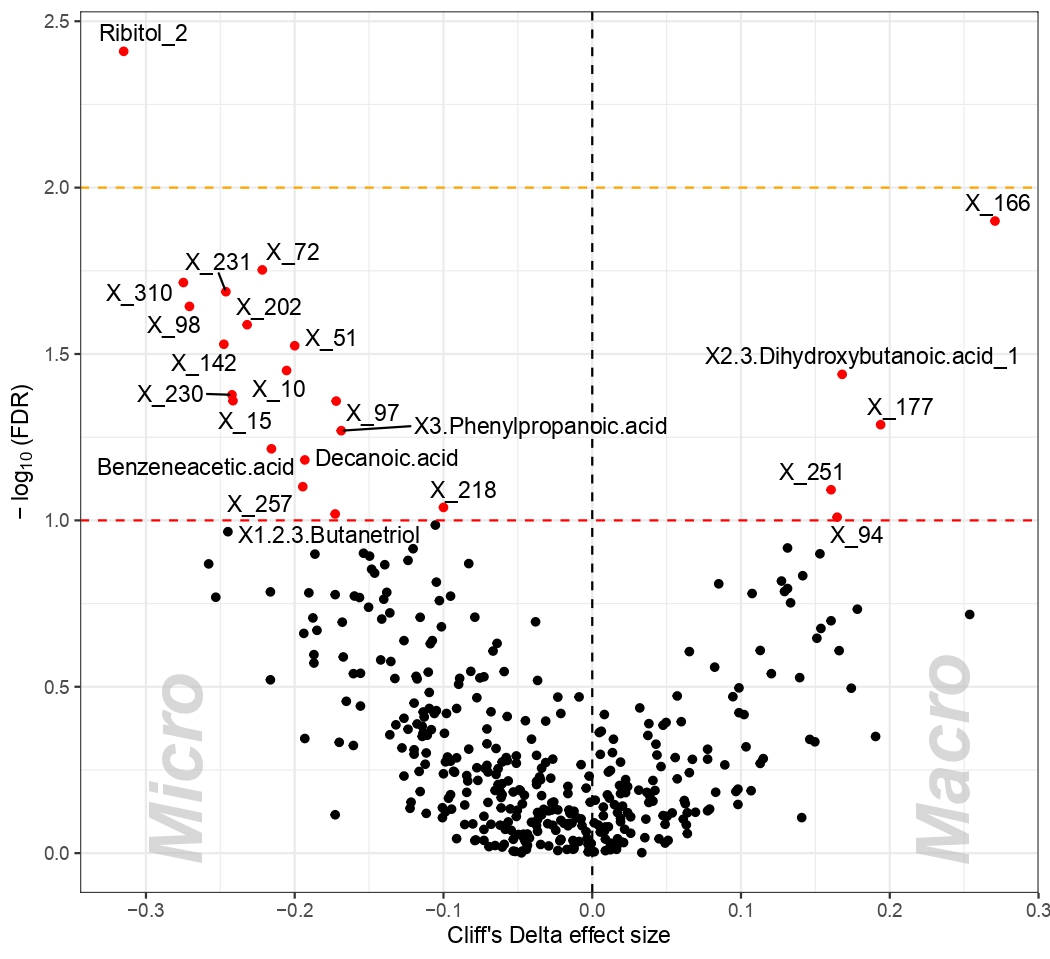


**Supplementary Figure 12**


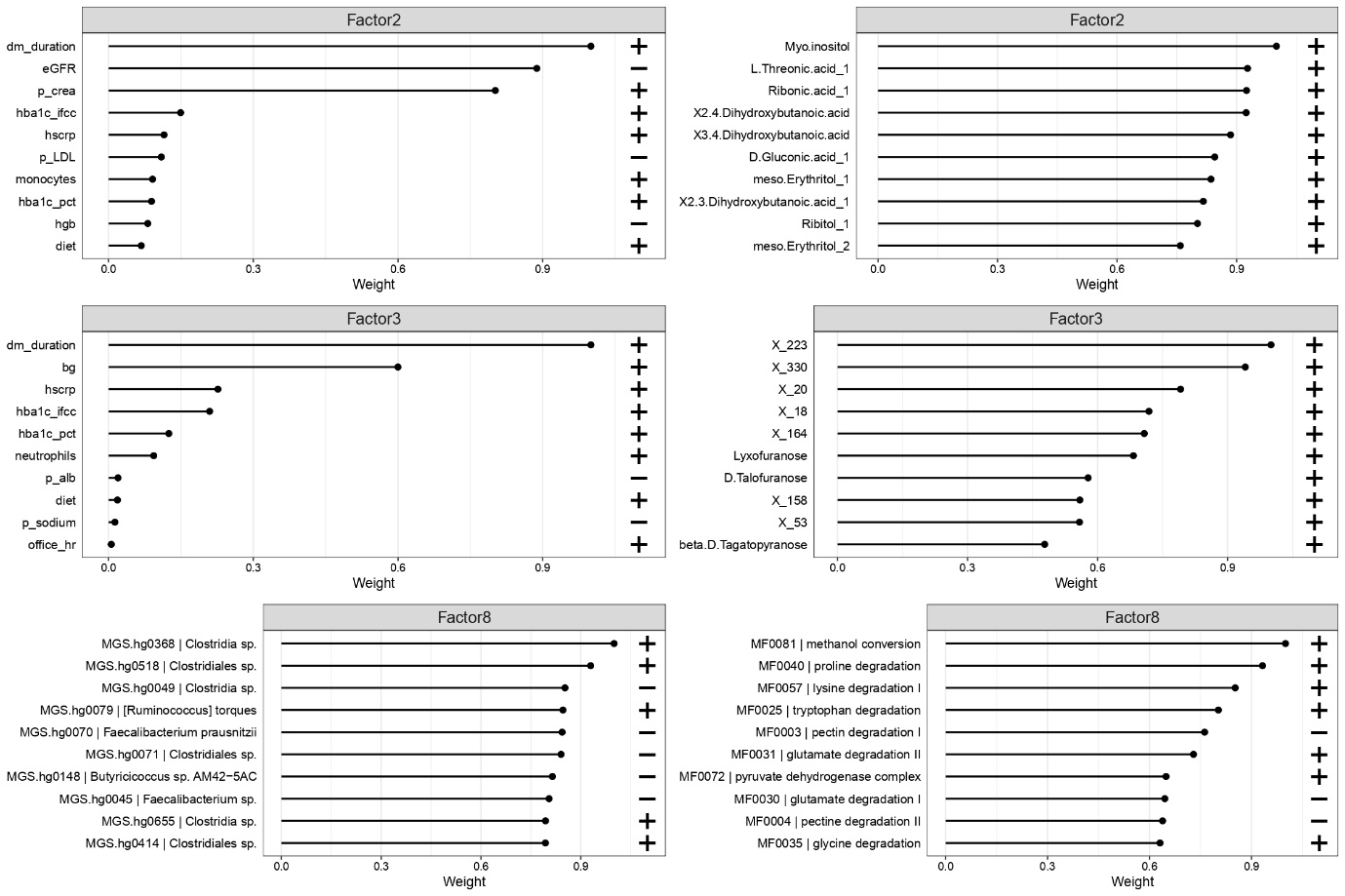


**Supplementary Figure 13**


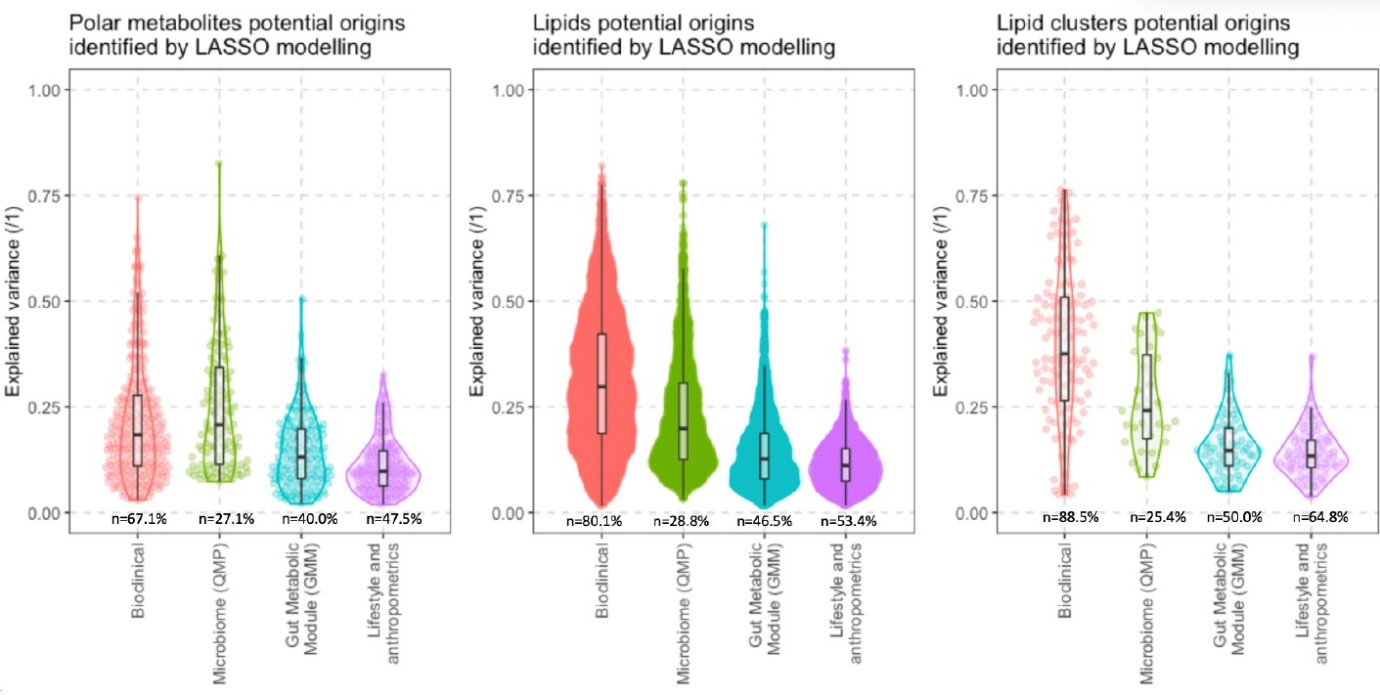


## **References**

1. S. A. Winther, P. Henriksen, J. K. Vogt, T. H. Hansen, L. Ahonen, T. Suvitaival, E. Hein Zobel, M. Frimodt-Møller, T. W. Hansen, T. Hansen, H. H. Parving, C. Legido-Quigley, P. Rossing, O. Pedersen, Gut microbiota profile and selected plasma metabolites in type 1 diabetes without and with stratification by albuminuria, *Diabetologia* **63**, 2713–2724 (2020).

2. R. Argelaguet, D. Arnol, D. Bredikhin, Y. Deloro, B. Velten, J. C. Marioni, O. Stegle, MOFA+: A statistical framework for comprehensive integration of multi-modal single-cell data, *Genome Biology* **21**, 1–17 (2020).

3. R. L. Klein, S. M. Hammad, N. L. Baker, K. J. Hunt, M. M. Al Gadban, P. A. Cleary, G. Virella, M. F. Lopes-Virella, Decreased plasma levels of select very long chain ceramide species Are associated with the development of nephropathy in type 1 diabetes, *Metabolism: Clinical and Experimental* **63**, 1287–1295 (2014).

4. S. Vieira-Silva, G. Falony, Y. Darzi, G. Lima-Mendez, R. Garcia Yunta, S. Okuda, D. Vandeputte, M. Valles-Colomer, F. Hildebrand, S. Chaffron, J. Raes, Species-function relationships shape ecological properties of the human gut microbiome, *Nature Microbiology* **1** (2016), doi:10.1038/nmicrobiol.2016.88.
